# Diet formulations with graded C:N ratios are associated with intestinal microbiota and health responses in GIFT tilapia (*Oreochromis niloticus*)

**DOI:** 10.3389/fmicb.2026.1918261

**Published:** 2026-10-08

**Authors:** Bocheng Huang, Xiangqin Lin, Shuyan Chi, Hongyu Liu, Shiwei Xie, Beiping Tan, Chao Ran, Zhigang Zhou, Junming Deng

**Affiliations:** 1 Laboratory of Aquatic Animal Nutrition and Feed, College of Fisheries, Guangdong Ocean University, Zhanjiang, China; 2 Institute of Feed Research, Chinese Academy of Agricultural Sciences, Beijing, China

**Keywords:** C:N ratio, GIFT (*Oreochromis niloticus*), intestinal health, intestinal microbiota, SCFAs

## Abstract

This research examined the effects of graded dietary carbon to nitrogen (C:N) ratios, by decreasing protein sources and increasing wheat starch and wheat bran, on growth performance, intestinal health, and intestinal microbiota in genetically improved farmed tilapia (GIFT; *Oreochromis niloticus*). Six experimental diets were formulated by adjusting dietary protein and carbohydrate to achieve C:N ratios of 7.34, 8.13, 9.20, 10.70, 12.15, and 14.47, and juvenile GIFT (initial mean body weight 5.30 g) were fed these diets for 58 days (6 groups, 3 replicates per group, 30 fish per cage). Growth performance indicators decreased significantly as the dietary C:N ratio increased, whereas the feed conversion ratio increased (*P* < 0.001). Diets with C:N ratios ≥ 10.7 were associated with more pronounced adverse responses, including impaired intestinal morphology, reduced trypsin activity, downregulated tight junction–related gene expression, decreased antioxidant enzyme activities, and enhanced inflammatory responses (*P* < 0.05). The graded diet formulations were associated with changes in intestinal microbial composition: higher C:N ratios were associated with positive linear trends in *Mycobacterium*, *Dietzia*, and Actinobacteriota abundance (*P* < 0.05). Parallel to these taxonomic shifts, short-chain fatty acid analysis showed significant among-diet differences in butyric acid levels (*P* < 0.05), with the highest numerical value observed in the CN10.7 treatment. Functional prediction indicated that higher C:N diets were associated with shifts in predicted microbial metabolic functions. Collectively, although diets with C:N ratios ≥ 10.7 were associated with predicted enrichment of carbohydrate-related microbial functions, these higher-ratio diets were also associated with impaired growth and intestinal health. Under the present experimental conditions and formulation strategy, the CN7.3–CN9.2 treatments showed less pronounced adverse host responses than the CN10.7–CN14.5 treatments, whereas further increases were associated with impaired growth, intestinal barrier function, and inflammatory and oxidative stress responses.

## 1 Introduction

Innovation in nutritional strategies has long been among the most effective approaches for improving growth performance in aquaculture species. However, previous research has primarily focused on host nutritional physiology, often overlooking the intestinal microbiota as a key regulatory target for enhancing growth-related traits. As a central component of the host “hologenome” (Limborg et al., 2018), the intestinal microbiota has a genetic capacity that far exceeds that of the host. For example, the non-redundant gene catalog of the grass carp intestinal microbiome comprises ∼ 576,000 genes (Li et al., 2024), compared with ∼27,000 protein-coding genes in the host genome (Wang Y. et al., 2015). This microbiota contributes to digestion, nutrient metabolism, and immune modulation (Enjalbert et al., 2017). Because microbial community composition is regulated by host genetics, environmental factors, and dietary intake (Ley et al., 2008; Li et al., 2012), dietary manipulation represents a feasible and practical strategy for intervention. Substantial evidence indicates that dietary protein level and source, energy content, and non-starch polysaccharides can modulate the intestinal microbiota (Ou et al., 2021), providing a scientific basis for microbiota-oriented optimization via precision nutrition to improve growth performance of farmed fish.

Research indicates that bacteria across diverse habitats, including intestinal, aquatic, and terrestrial systems, display similar elemental stoichiometry, with C:N ratios spanning between 3.29 and 6.05 (Mouginot et al., 2014; Reese et al., 2018; Zimmerman et al., 2014). Accordingly, based on a microbial carbon use efficiency of 0.3 (Sinsabaugh et al., 2013), the required C:N ratio is estimated to range from 11.3 to 20.2, consistent with optimal biofloc development and improved growth and health in crucian carp (*Carassius auratus*) and African catfish (*Clarias gariepinus*) when the water C:N ratio was maintained between 15 and 20 (Dauda et al., 2018; Wang G. et al., 2015). Nevertheless, typical aquafeeds are formulated to meet animals’ dietary requirements and often contain lower C:N ratios (∼7–10). Consequently, studies in *Litopenaeus vannamei* and *Cyprinus carpio* suggest that such formulations do not provide sufficient carbon to meet these stoichiometric requirements, necessitating carbohydrate supplementation to raise the dietary C:N ratio (Guo et al., 2020; Zhao et al., 2014).

Direct studies linking dietary C:N ratio to intestinal responses in aquatic animals remain scarce. In *Litopenaeus vannamei*, increasing the C:N ratio of feed input by sucrose supplementation altered the intestinal microbiota and metabolome, although these responses occurred concurrently with biofloc formation and changes in the rearing microbial environment (Guo et al., 2020). More recently, dietary C:N manipulation in *Oncorhynchus mykiss* has been shown to affect water chemistry and microbial activity in recirculating aquaculture systems, but intestinal responses were not evaluated (Huang et al., 2024). Related nutritional studies using dietary protein-to-carbohydrate ratios have demonstrated effects on intestinal digestive function, microbial composition, and host metabolic responses in *Sparus aurata*, *Oreochromis niloticus*, and *Apostichopus japonicus* (Basto-Silva et al., 2022; Liu et al., 2025; Ge et al., 2026). Thus, the direct effects of graded dietary C:N ratios on fish intestinal microbiota and intestinal health remain poorly characterized.

Such stoichiometric disparities between dietary supply and microbial demand may function as a metabolic switch that reshapes the functional priorities of the intestinal microbiota. When fermentable carbohydrates are limited, the intestinal microbiota relies more heavily on dietary proteins for energy production, thereby promoting proteolytic fermentation (Villasante et al., 2019). This shift can lead to inefficient nitrogen utilization and the accumulation of potentially detrimental metabolic byproducts (e.g., phenolic compounds and ammonia), which impair intestinal mucosal barrier function and trigger localized inflammation (Villasante et al., 2022). In contrast, increasing dietary C:N ratio by incorporating non-structural carbohydrates promotes microbial resource allocation toward saccharolytic fermentation, thereby increasing the synthesis of short-chain fatty acids (SCFAs). These SCFAs promote intestinal homeostasis through the reduction of luminal pH, the inhibition of opportunistic pathogens, and the provision of energy substrates for epithelial cells (Gratz et al., 2019). Moreover, higher dietary C:N ratios may induce a protein-sparing effect, improving nitrogen retention and reducing waste discharge, thereby supporting more sustainable aquaculture practices (Huyben et al., 2020). Nevertheless, how graded dietary C:N ratios reshape intestinal microbial metabolism and interact with host intestinal health remains insufficiently understood, particularly in fish. Although adjusting this ratio may provide an effective strategy to modulate the microbiota and improve host health, the associated increase in dietary carbohydrate necessitates an experimental model with high dietary adaptability.

Nile tilapia (*O. niloticus*), which is among the most widely produced fish globally (FAO, 2024), exhibits broad dietary adaptability and relatively high tolerance to carbohydrate-rich diets (Chen et al., 2020). Accordingly, genetically improved farmed tilapia (GIFT; *O. niloticus*) was used as the experimental model in this study. Diets with graded C:N ratios were formulated based on a commercial feed recipe to elicit distinct intestinal microbial responses, and the associations among intestinal microbiota, growth performance, and intestinal health were systematically evaluated.

## 2 Materials and methods

### 2.1 Experimental diets

Six experimental diets for juvenile tilapia were prepared to provide a gradient of dietary C:N ratios. A commercial tilapia feed formulation served as the baseline, and the highest-C:N diet was formulated to achieve a C:N ratio approximately twice that of the baseline diet. The four intermediate diets were formulated by increasing wheat starch and wheat bran and decreasing protein sources in equal stepwise increments between these two endpoint formulations. Based on the analyzed C:N ratios (7.34, 8.13, 9.20, 10.70, 12.15, and 14.47), the diets were designated CN7.3, CN8.1, CN9.2, CN10.7, CN12.2, and CN14.5. The analyzed C:N ratios were not evenly spaced because the arithmetic sequence was applied to the inclusion levels of feed ingredients rather than directly to the C:N ratios.

The ingredients were ground and screened using a 60-mesh screen, and then measured out based on the formulations. Micro-ingredients were premixed using a stepwise enlargement method and subsequently blended with macro-ingredients in a V-type vertical mixer until a homogeneous mixture was achieved. Lipids were incorporated by hand-mixing, after which distilled water (30%, w/w) was added. A twin-screw extruder was employed to process the resulting mixture into pellets with a 2.5-mm diameter. Pellets were air-dried for 36 h and packed in sealed bags. Ingredients and proximate composition are presented in Table 1.

**TABLE 1:** Ingredients and proximate composition (% dry weight) of experimental diets.

Ingredients	Diets					
	CN7.3	CN8.1	CN9.2	CN10.7	CN12.2	CN14.5
Fish meal	6.00	5.00	4.00	3.00	2.00	1.00
Chicken meal	5.00	4.00	3.00	2.00	1.00	0.00
Soybean meal	16.00	13.00	10.00	7.00	4.00	1.00
Soy protein concentrate	5.00	4.00	3.00	2.00	1.00	0.00
Canola meal	18.00	16.00	14.00	12.00	10.00	8.00
Cottonseed meal	18.00	16.00	14.00	12.00	10.00	8.00
Wheat starch	16.72	17.72	18.72	19.72	20.72	21.72
Wheat bran	8.00	17.00	26.00	35.00	44.00	53.00
Soybean oil	3.50	3.50	3.50	3.50	3.50	3.50
Soybean lecithin	1.00	1.00	1.00	1.00	1.00	1.00
Mineral premix^1^	0.50	0.50	0.50	0.50	0.50	0.50
Vitamin premix^2^	0.20	0.20	0.20	0.20	0.20	0.20
Vitamin C	0.03	0.03	0.03	0.03	0.03	0.03
Choline chloride	0.30	0.30	0.30	0.30	0.30	0.30
Ca(H_2_PO_4_)_2_	1.50	1.50	1.50	1.50	1.50	1.50
Sodium chloride	0.20	0.20	0.20	0.20	0.20	0.20
Y_2_O_3_	0.05	0.05	0.05	0.05	0.05	0.05
Total	100.00	100.00	100.00	100.00	100.00	100.00
Proximate composition						
Dry matter (%)	90.63	90.52	90.80	90.62	90.59	90.65
Crude protein (DM, %)	37.78	34.28	30.10	26.42	22.72	19.04
Crude lipid (DM, %)	6.13	6.30	6.20	6.18	6.09	6.23
Organic matter^3^ (DM, %)	92.36	92.48	92.77	92.95	93.33	93.42
Crude fiber (DM, %)	5.44	6.31	7.02	7.75	8.16	8.54
NFE^4^ (DM, %)	43.01	45.59	49.45	52.60	56.36	59.61
Crude fiber/NFE	0.13	0.14	0.14	0.15	0.14	0.14
C/N ratio	7.34	8.13	9.20	10.70	12.15	14.47

^1^Mineral premix (mg kg^–1^mixture): Fe, 28,000–150,000; Cu, 560–5,000; Zn 7,000–30,000; Mn, 3,500–20,000; I, 70–400; Se, 40–100; Co, 30–400.

^2^Vitamin premix guaranteed per kg: Vitamin A, 6,000,000 IU; Vitamin D_3_, 1,250,000 IU; Vitamin E, 40,000 mg; Vitamin K_3,_ 6,000 mg; Vitamin B_1,_ 3,040 mg; Vitamin B_2_, 8,000 mg; Vitamin B_6_, 10,000 mg; Vitamin B_12_, 13 mg; Nicotinamide, 26,270 mg; Calcium Pantothenate, 15,000 mg; Folic Acid, 590 mg; Biotin, 25 mg; Vitamin C (as ascorbic acid equivalent), 60,000 mg; Inositol, 1,500 mg.

^3^Organic matter = 100% − crude ash.

^4^NFE (nitrogen-free extract) = 100% − (crude protein + crude lipid + crude ash + crude fiber).

### 2.2 Fish and experimental conditions

Juvenile GIFT were procured from a local breeding facility, and acclimated for 6 days in six of 18 synthetic fiber cages (0.8 m × 1.2 m × 1.0 m) placed in a 5 m × 5 m × 1.5 m concrete pond at the Freshwater Research Center of Guangdong Ocean University. After acclimation, fish were deprived of feed for 24 h and healthy individuals of uniform size were randomly distributed among the 18 cages, with three replicate cages assigned to each treatment. The initial mean body weight was 5.30 ± 0.003 g. Each treatment had three replicates, with one synthetic fiber cage per replicate and 30 fish per cage. The cages assigned to different dietary treatments were randomly positioned within the pond. Experimental groups were fed with the corresponding diet to apparent satiation twice daily.

The pond was covered with three-needle sunshade nets positioned 1.8 m above the water surface to reduce direct sunlight, and continuous aeration was provided by blowers. Approximately 20% of the water was replaced every 2 days. Water quality was monitored regularly, with dissolved oxygen maintained above 6.0 mg/L, ammonia nitrogen below 0.4 mg/L, and water temperature between 26.0 °C and 30.0 °C. The feeding trial lasted for 58 days.

### 2.3 Sample collection

At the end of the feeding trial, all fish were fasted for 24 h prior to sampling. Fish number and total biomass per cage were recorded to evaluate growth performance and feed utilization. Mean initial and final body weight per cage were used to calculate weight gain (WG) and specific growth rate (SGR), whereas total feed intake and total biomass gain per cage were used to calculate feed conversion ratio (FCR) and feed intake ratio (FIR). Fish were anesthetized with eugenol (1:10,000) before handling.

Three fish per cage were randomly collected for measuring the length of body and intestine, along with the weight of body, viscera, and liver, to calculate morphology indices. For blood sampling, eight fish were randomly selected from each cage. Blood samples were collected separately for different analyses. One portion was collected from the caudal vein using untreated syringes, whereas another portion, intended for plasma preparation, was collected using syringes pre-rinsed with heparin solution (1,250 U/mL). Plasma was subsequently obtained by centrifugation. After centrifugation at 3,000 rpm for 10 min at 4 °C, the resulting plasma from each cage was pooled to obtain one cage-level sample for subsequent analyses.

For intestinal sampling, the proximal intestine and part of the distal intestine from six fish per cage were transferred into cryogenic tubes, flash-frozen with liquid nitrogen, and stored at −80 °C for homogenate preparation. For enzyme and antioxidant analyses, intestinal tissues from the six fish within each cage were physically pooled before homogenization, yielding one cage-level sample. The remaining distal intestinal tissues from the six fish within each cage were physically pooled before RNA extraction for qPCR analysis. Two distal intestinal segments from two fish within each cage were fixed separately, one in 4% paraformaldehyde for histological analysis and the other in 2.5% glutaraldehyde for scanning electron microscopy.

After sampling, remaining fish were maintained for 2 days, then fed to satiation. At 6 h post-feeding, distal intestinal contents were collected from four fish per cage, pooled, and stored in liquid nitrogen and then at −80 °C for subsequent analyses. The pooled intestinal-content sample from each cage was used as one cage-level biological replicate for microbiota and SCFA analyses.

For all endpoints, the cage was considered the experimental unit, with three independent cage-level biological replicates per treatment.

### 2.4 Analyses

#### 2.4.1 Proximate composition

The proximate composition analysis of experimental diets conformed to the standard methods of the Association of Official Analytical Chemists (AOAC, 1990), 15th edition. Crude lipid was determined by Soxhlet extraction (AOAC 920.39), crude ash was assessed by carbonization and subsequent ignition at 550 °C for 6 h (AOAC 923.03), and moisture was measured by drying to constant weight at 105 °C (AOAC 934.01). Carbon and nitrogen contents were measured using an EA-ISO Link elemental analyzer (Thermo Fisher Scientific, Waltham, MA, USA), and were used to calculate dietary C:N ratio and crude protein.

#### 2.4.2 Biochemical analysis

The cage-level intestinal and plasma samples described in Section “2.3 Sample collection” were used for biochemical analyses. Intestinal tissue (approx. 0.2 g) was homogenized on ice in nine volumes (w/v) of 0.9% physiological saline. Homogenates were centrifuged at 2500 r/min for 8 min at 4 °C, and supernatants were harvested. Total protein content was determined, and all samples were adjusted to the same protein concentration with physiological saline.

Biochemical parameters were determined using commercial kits according to manufacturers’ instructions. Kits from Nanjing Jiancheng Bioengineering Institute (Nanjing, China) were used to determine total protein (TP), total antioxidant capacity (T-AOC), total superoxide dismutase (T-SOD), reduced glutathione (GSH), glutathione peroxidase (GSH-Px), catalase (CAT), malondialdehyde (MDA), α-amylase (AMS), and lipase.

Commercial ELISA kits (Shanghai Enzyme-linked Biotechnology Co., Ltd., Shanghai, China) were used to determine intestinal trypsin activity and plasma diamine oxidase (DAO) activity, along with plasma concentrations of D-lactic acid (D-LA), lipopolysaccharide (LPS), and endothelin (ET).

#### 2.4.3 Intestinal morphology

Tissues were transferred and dehydrated through ethanol after fixation, cleared in xylene, embedded in paraffin, and sectioned at 5 μm. Sections were mounted, dried, stained with hematoxylin and eosin (H&E), and sealed. Briefly, paraffin sections were deparaffinized in xylene and rehydrated through a graded ethanol series to distilled water. The sections were stained with hematoxylin, rinsed with running tap water, differentiated in acid alcohol, and then blued in tap water. Subsequently, the sections were counterstained with eosin, dehydrated through graded ethanol, cleared in xylene, and mounted with neutral resin. Images were captured using a light microscope (Nikon Eclipse Ni-U, Japan). Histological tissues were not physically pooled. Ten measurements of intestinal villus and muscularis parameters were performed on each histological section. Multiple measurements acquired from one section served as within-section subsamples. We first averaged these subsample values to obtain a single value for each cage. All subsequent statistical analyses were carried out based on three independent cage-level biological replicates in each treatment.

For scanning electron microscopy, samples were fixed with osmium tetroxide, rinsed with buffer, dehydrated through graded ethanol, and exchanged with isoamyl acetate. Samples were dried, sputter-coated, then mounted on stubs with conductive adhesive, and observed using a scanning electron microscope (HITACHI Regulus 8100, Tokyo, Japan).

#### 2.4.4 Gene expression analysis

Total RNA was extracted using a commercial kit (Quanshijin Biotechnology Co., Ltd., Beijing, China) according to the manufacturer’s instructions. RNA concentration was measured using a Nanodrop 2000 spectrophotometer (Thermo Fisher Scientific, Waltham, MA, USA) and diluted to 800 ng/μL. cDNA was synthesized using a reverse transcription kit (Aikeri Bioengineering Co., Ltd., Changsha, China). Quantitative real-time PCR was performed using SYBR^®^ Green Premix Pro Taq HS qPCR Kit II (Aikeri Bioengineering Co., Ltd., Changsha, China) following the previously described conditions (Huang et al., 2022) on a LightCycler 480 II system (Roche/LightCycler, Germany). *β-actin* was selected as the reference gene because it is one of the commonly used internal controls in fish qPCR studies, including studies in Nile tilapia, and has previously been applied in intestinal gene-expression analyses of this species (Miao et al., 2020; Reda et al., 2022). However, no additional candidate reference genes were evaluated in the present study. Relative expression was calculated using the 2^–ΔΔCt^ method (Livak and Schmittgen, 2001). Primer sequences are listed in Table 2. The amplification efficiencies were determined using representative cDNA templates serially diluted by 5-fold, 25-fold, and 125-fold. Standard curves were generated by plotting Ct values against the logarithm of the relative template concentration, and amplification efficiency was calculated. For analysis, *n* = 3 cage-level biological replicates per treatment, as described in Section “2.3 Sample collection”; duplicate wells were used as technical replicates.

**TABLE 2:** Forward and reverse primers for real-time quantitative PCR.

Target gene	Primer direction	Primer sequence (5′–3′)	Accession number	Amplification efficiencies
*occludin*	F	GGAGGAAAGCCGCAGTGTTCAG	XM_025899615.1	97.89%
	R	GTCGTAGGCATCGTCATTGTAGGAG		
zo-1	F	ACATCGTGCGCTCCAACCAT	XM_013270540.3	92.03%
	R	GGCTGGACTGTGCTTGTGGT		
zo-3	F	GACCGCCTCCAGATGATTCC	XM_013267128.3	107.74%
	R	AATCCCAGACCTGATGGTGC		
claudin	F	GTCTGTTTCTGGGCGTGGTGTC	XM_019367708.2	104.63%
	R	ACTCCGACTGACTCCTCATCTTCC		
claudin-11	F	CATCCAGACTACTCGTGCCC	XM_003442886.4	98.89%
	R	GCTCTGGGGTTCATTCCCAA		
claudin-12	F	CGCAGTGGCGTATAACGAAA	XM_005472700.3	101.32%
	R	CAACTTGGAGTACCACTCTGAA		
tnf-α	F	TAGAAGGCAGCGACTCAA	NM_001279533.1	108.99%
	R	CCTGGCTGTAGACGAAGT		
il-1β	F	GACAGCCAAAAGAGGAGC	XM_019365844.2	99.62%
	R	TCTCAGCGATGGGTGTAG		
il-6	F	ATAGCAAGCATCTACACGCATCTCC	XM_003453898.2	107.60%
	R	GGGCTGCCAGGGAATTGTAAGTC		
il-8	F	GCACTGCCGCTGCATTAAG	NM_001279704.1	100.29%
	R	GCAGTGGGAGTTGGGAAGAA		
il-10	F	GAGATGTCACCCAGTGTAGGAA	XM_013269189.3	95.25%
	R	TGTGTCGTTTAGAAGCCAGGT		
tgf-β1	F	CTGGCTCCAAGGGAATGAGAAT	XM_039622897.1	95.78%
	R	CCCTCCTGCTCATAGTCCCA		
β-actin	F	CCACACAGTGCCCATCTACGA	XM_003443127.5	104.94%
	R	CCACGCTCTGTCAGGATCTTCA		

zo-1, zonula occludens 1; zo-3, zonula occludens 3; tnf-α, tumor necrosis factor α; il-1β, interleukin-1β; il-6, interleukin 6; il-8, interleukin 8; il-10, interleukin 10; tgf-β1, transforming growth factor β1.

#### 2.4.5 Microbiota analysis

Genomic DNA from intestinal contents was isolated using a VAMNE Stool/Soil DNA Extraction Kit (Vazyme, China). DNA concentration was quantified using 1× dsDNA HS Working Solution (Yeasen, Shanghai, China) with a Synergy HTX multi-mode reader (BioTek, Santa Clara, CA, USA). The V3–V4 region of the bacterial 16S rRNA gene was amplified using the primer pair F: ACTCCTACGGGAGGCAGCA and R: GGACTACHVGGGTWTCTAAT. PCR was performed in two rounds. Round 1 PCR was conducted using KOD FX Neo (No. KFX-201S, Toyobo, Osaka, Japan) following the manufacturer’s optimized protocol, and amplicons were purified with VAHTS DNA Clean Beads (Vazyme, Nanjing, China). PCR negative controls, specifically no-template controls, were included during the amplicon amplification step. No amplicon products were obtained from these negative controls; therefore, they were not included in library construction or sequencing and are not shown in the sequencing results. Round 2 PCR was conducted using Q5 High-Fidelity PCR Mix (New England Biolabs, Ipswich, MA, USA). Libraries were quantified and then sequenced on an Illumina NovaSeq 6000 platform.

Raw data were first quality-filtered using Trimmomatic (Trimmomatic, RRID: SCR_011848, version 0.33) (Bolger et al., 2014). Subsequently, primer sequences were identified and removed via Cutadapt (Martin, 2011) (Cutadapt, RRID: SCR_011841, version 1.9.1^1^), with a maximum mismatch rate of 20% and minimum primer coverage of 80%, to obtain clean reads. Paired-end reads obtained from the previous steps were assembled using USEARCH (USEARCH, RRID: SCR_027438, version 10.0), with a minimum overlap length of 10 bp, a minimum similarity of 90% within the overlapping region, and a maximum of 5 mismatches. Chimeric sequences were removed using UCHIME (UCHIME, RRID: SCR_008057, version 8.1). The non-chimeric reads generated from the above steps were then processed using the DADA2 package (DADA2, RRID: SCR_023519, version 1.18^2^) method in QIIME2 (QIIME2, RRID: SCR_008249, version 2020.06^3^) to denoise sequences and generate amplicon sequence variants (ASVs). ASVs with a total abundance of fewer than two reads were removed before downstream analyses.

Taxonomic identification was also performed using QIIME2 based on representative ASV sequences. Sequences were aligned against the SILVA database (SILVA, RRID: SCR_006423, Release 138^4^). Taxonomy was assigned using a combined BLAST- and Naive Bayes classifier-based method. Briefly, representative ASV sequences were first classified against the reference database using the classify-consensus-blast method in QIIME2; sequences that could not be matched to the reference database were further classified using classify-sklearn. The parameters for taxonomic assignment were a minimum sequence similarity of 90%, minimum coverage of 90%, minimum consensus of 51%, and classifier confidence of 0.7.

Phylum- and genus-level taxonomic profiles were summarized as relative abundances. These relative-abundance data were used directly for subsequent taxon-level statistical analyses without log-ratio or other compositional transformation.

Alpha diversity indices were subsequently quantified via the annotated results using QIIME2. Beta diversity was calculated based on Bray–Curtis, binary Jaccard, and weighted and unweighted UniFrac distance matrices. For alpha-diversity and Bray–Curtis/binary Jaccard analyses, the filtered ASV count table was used directly without rarefaction or other library-size normalization. For Bray–Curtis/PERMANOVA, the effective library size of each sample was defined as the sum of ASV counts in the final filtered ASV count matrix. To assess whether variation in effective library size influenced the PERMANOVA result, two sensitivity analyses were performed using the same ASV matrix: Bray–Curtis/PERMANOVA after total-sum scaling to relative abundance, and Bray–Curtis/PERMANOVA after rarefying all samples without replacement to the minimum effective library size. Principal coordinates analysis (PCoA) was used for beta diversity ordination. PERMANOVA (Adonis) analysis was used to test whether beta diversity differed among groups using 9,999 unrestricted permutations. For the Bray–Curtis matrix, multivariate dispersion was assessed using the betadisper function in the R package vegan (type = centroid), and significance was tested using permutest with 9,999 unrestricted permutations.

Functional profiles of the microbial communities were predicted using PICRUSt2 (PICRUSt2, RRID:SCR_022647, Phylogenetic Investigation of Communities by Reconstruction 2) (Douglas et al., 2020), which infers metagenomic functional potential from 16S rRNA gene sequences based on phylogenetic placement and reference genome databases. In addition, microbial metabolic function was annotated using FAPROTAX (Functional Annotation of Prokaryotic Taxa) (Louca et al., 2016) by mapping taxonomic information to curated functional categories derived from literature-supported microbial metabolic traits.

#### 2.4.6 SCFA analysis

Intestinal contents were weighed and homogenized with ultrapure water and glass beads using a tissue grinder (MB-96; Zhejiang Meibi Instrument Co., Ltd., Jiaxing, China), and centrifuged to recover the supernatants. A total of 200 μL supernatant were then acidified with 20 μL of 375 μg/mL 4-methylvaleric acid (internal standard; Sigma-Aldrich, Shanghai, China), extracted with 280 μL ether (Titan, Shanghai, China), vortexed (QL-866, Haimen Kylin-bell, Haimen, China), and centrifuged again under the same conditions. The organic phase was analyzed by GC-MS (Agilent 7890B coupled with a 5977B mass selective detector, Agilent Technologies, Santa Clara, CA, USA). GC-MS instrumental analysis was performed following the method described by Xue et al. (2018). Briefly, separation was carried out on a J&W Scientific HP-FFAP capillary column (30 m × 0.25 mm × 0.25 μm, Agilent Technologies). A volume of 1 μL sample was injected in split mode (split ratio 20:1) at an inlet temperature of 175 °C. Helium (99.999% purity) was used as carrier gas at a constant flow rate of 1 mL/min. The oven temperature program was set as follows: initial temperature 90 °C, increased to 150 °C at 12 °C/min, then ramped to 220 °C at 20 °C/min and held for 4.5 min. The transfer-line and electron-impact (EI) ion-source temperatures were 220 °C and 230 °C, respectively, with a solvent-delay time of 3 min.

The SCFAs were identified by GC-MS according to their specific retention time and typical ions. On the day of analysis, we diluted the mixed standard solutions to concentrations of 1, 2.5, 5, 7.5, 10, 25, 50, 80, 100, 150 and 300 μL/L for each acid with ether and made 5 replicates for every concentration. We used the TIC mode to obtain typical ions with a special mass-to-charge ratio of every SCFA and then used a SIM mode to collect information with typical ions. A standard curve was made for each SCFA based on the data obtained by the SIM mode. Limits of detection (LOD) and quantification (LOQ) were determined by considering the peak area relative to 3 and 10 times the signal-to-noise ratio, respectively. SCFA concentrations in the samples were calculated from the corresponding calibration curves and expressed as μg/g.

### 2.5 Data calculation and statistical analysis

Indices were calculated as follows:


$$\mathrm{Weight}\,\mathrm{gain}\,\left(\mathrm{WG}\right)=\left(W_{t}-W_{i}\right)/W_{i}$$

$$\mathrm{Specific}\mathrm{growth}\mathrm{rate}\left(\mathrm{SGR},\%/\mathrm{day}\right)=100\times \left(\ln W_{t}-\ln W_{i}\right)/T$$

$$\mathrm{Feed}\,\mathrm{conversion}\,\mathrm{ratio}\,\left(\mathrm{FCR}\right)=F_{i}/\left(B_{t}-B_{i}\right)$$

$$\mathrm{Feed}\mathrm{intake}\mathrm{ratio}\left(\mathrm{FIR},\%/\mathrm{day}\right)=100\times F_{i}/\left[T\times \left(B_{t}+B_{i}\right)/2\right]$$

$$\mathrm{Condition}\,\mathrm{factor}\,\left(\mathrm{CF},g/{\mathrm{cm}}^{3}\right)=100\times W_{t}/L^{3}$$

$$\mathrm{Viscerosomatic}\mathrm{index}\left(\mathrm{VSI},\%\right)=100\times W_{v}/W_{t}$$

$$\mathrm{Hepatosomatic}\mathrm{index}\left(\mathrm{HSI},\%\right)=100\times W_{l}/W_{t}$$

$$\mathrm{Relative}\mathrm{intestine}\mathrm{length}\left(\mathrm{RIL},\%\right)=100\times L_{i}/L$$

where W_i_ and W_t_ are initial and final body weight (g), T is trial duration (days), B_i_ and B_t_ are initial and final total cage biomass (g), F_i_ is total feed intake (g), L is body length (cm), W_1_ is liver weight (g), W_v_ is visceral weight (g), and Lá is intestine length (cm).

Unless otherwise specified, univariate data were analyzed by one-way analysis of variance (ANOVA) after testing normality and homogeneity of variance. Analyses were performed using SPSS 21.0 (*SPSS*, RRID: SCR_002865, SPSS Inc., Chicago, USA). When significant differences were detected (*P* < 0.05), Duncan’s multiple range test was used for *post hoc* comparisons. Additionally, linear and quadratic trends across the six ordered dietary treatments were evaluated using orthogonal polynomial contrasts within the one-way ANOVA framework. The treatments CN7.3, CN8.1, CN9.2, CN10.7, CN12.2, and CN14.5 were treated as six equally spaced ordered levels. First- and second-degree orthogonal polynomial contrasts were used to test the linear and quadratic components, respectively. These contrasts therefore evaluated trends across the ordered dietary treatments rather than regression per unit increase in the measured C:N ratio.

For microbial taxonomic and predicted functional profiles, primary statistical analyses were performed directly on untransformed relative-abundance data. Features with an overall mean relative abundance >0.1% were included in multiple-testing correction. Benjamini–Hochberg false discovery rate (BH-FDR) correction was performed separately for phylum-level taxa, genus-level taxa, PICRUSt2-predicted functions/pathways, and FAPROTAX-predicted functions; these four data domains were not combined into a single correction family. Within each data domain, *P*-values from one-way ANOVA, linear trend analysis, and quadratic trend analysis were adjusted as separate testing families. FDR-adjusted *P*-values < 0.05 were considered statistically significant.

To assess the robustness of the primary relative-abundance findings against potential compositional effects, an additional compositional sensitivity analysis was performed using ANCOM-BC2. ANCOM-BC2 was run using the corrected final ASV count matrix as the lowest-level data input for bias estimation. Phylum- and genus-level count matrices aggregated from the same ASV table were filtered using the overall mean relative-abundance > 0.1% criterion and supplied as aggregate data for target-level inference. Differential abundance was assessed using global tests, linear trend tests, and quadratic trend tests. For consistency with the primary relative-abundance trend analyses, linear and quadratic trend terms in ANCOM-BC2 were defined using the same first- and second-degree orthogonal polynomial coding across the six ordered treatment levels (CN7.3–CN14.5), respectively. BH-FDR correction was applied separately within each rank-by-test family (phylum/global, phylum/linear, phylum/quadratic, genus/global, genus/linear, and genus/quadratic). ANCOM-BC2-derived multiple-testing adjusted *P*-values (*q*-values) < 0.05 were considered statistically significant, and pseudo-count sensitivity analysis was enabled.

## 3 Results

### 3.1 Growth performance and morphology

The FBW, WG, and SGR generally decreased as dietary C:N ratios increased (*P* < 0.001, Table 3). Specifically, these indices were significantly higher in the CN7.3 group than in the other groups, whereas the lowest values occurred in the CN14.5 group. Similarly, CF decreased as dietary C:N ratios increased and was significantly lower in fish fed CN14.5 than in fish fed CN7.3 and CN8.1. In contrast, FIR and FCR generally increased with increasing dietary C:N ratio and were significantly higher in the CN12.2 and CN14.5 groups than in the CN7.3 and CN8.1 groups (*P* < 0.001). Regarding morphological indices, HSI and VSI increased significantly under higher C:N ratios (*P* < 0.001), whereas SR (survival rate) and RIL did not differ among treatments (*P* > 0.05). Additionally, FBW, WG, CF and VSI showed significant linear trends across the ordered dietary treatments (*P* < 0.001), while SGR, FIR, FCR and HSI exhibited both significant linear and quadratic trends (*P* < 0.05).

**TABLE 3:** Effects of dietary C:N ratios on the growth performance and morphological indices of GIFT.

Items	Groups						SEM	*P*-value		
	CN7.3	CN8.1	CN9.2	CN10.7	CN12.2	CN14.5		ANOVA	Linear	Quadra
FBW (g)	112.44^f^	102.96^e^	91.88^d^	82.71^c^	72.48^b^	60.74^a^	4.276	<0.001	<0.001	0.529
WG (fold)	20.21^f^	18.51^e^	16.36^d^	14.62^c^	12.68^b^	10.49^a^	0.809	<0.001	<0.001	0.518
SGR (%/day)	5.27^f^	5.12^e^	4.92^d^	4.74^c^	4.51^b^	4.21^a^	0.087	<0.001	<0.001	0.008
FIR (%/day)	3.41^a^	3.57^ab^	3.91^b^	4.27^c^	4.93^d^	5.20^d^	0.163	<0.001	<0.001	0.022
FCR	1.06^a^	1.12^a^	1.25^b^	1.38^c^	1.61^d^	1.74^e^	0.061	<0.001	<0.001	0.005
SR (%)	98.89	98.89	100.00	100.00	100.00	100.00	0.254	0.571	0.124	0.464
CF (g/cm^3^)	3.42^cd^	3.57^d^	3.30^bc^	3.18^abc^	3.06^ab^	3.00^a^	0.044	<0.001	<0.001	0.593
VSI (%)	9.43^a^	8.90^a^	8.89^a^	9.69^a^	10.96^b^	12.06^b^	0.067	<0.001	<0.001	0.108
HSI (%)	1.20^a^	1.27^a^	1.11^ab^	1.59^bc^	1.90^cd^	1.97^d^	0.218	<0.001	<0.001	<0.001
RIL (%)	754.54	793.07	768.67	766.94	748.40	766.39	14.517	0.972	0.806	0.816

Different superscript letters within a row indicate significant differences (*P* < 0.05, *n* = 3 cage-level means). FBW, final body weight; WG, weight gain; SGR, specific growth rate; SR, survival rate; FCR, feed conversion ratio; FIR, feed intake ratio; CF, condition factor; VSI, viscerosomatic index; HSI, hepatosomatic index; RIL, relative intestine length.

### 3.2 Intestinal morphology

Muscularis thickness, villus width, villus height, and crypt depth generally decreased as dietary C:N ratios increased (Table 4). Specifically, fish fed CN7.3 and CN8.1 diets exhibited significantly higher muscularis thickness, villus width, villus height, and crypt depth than the CN12.2 and CN14.5 groups, and a significantly higher villus height-to-crypt depth ratio than the CN12.2 and CN14.5 groups (*P* < 0.05). Overall, the lowest values were observed in the high C:N treatments (CN12.2–CN14.5), with villus height and crypt depth reaching their minimum in the CN14.5 group. Additionally, muscularis thickness, villus width, villus height, crypt depth, and villus height-to-crypt depth ratio all showed significant linear trends across the ordered dietary treatments (*P* ≤ 0.001), whereas muscularis thickness, and villus width further exhibited significant quadratic trends (*P* < 0.05).

**TABLE 4:** Effects of dietary C:N ratios on the distal intestinal morphology of GIFT.

Items	Groups						SEM	*P*-value		
	CN7.3	CN8.1	CN9.2	CN10.7	CN12.2	CN14.5		ANOVA	Linear	Quadratic
MT (μm)	57.65^e^	55.30^de^	51.46^cd^	48.98^bc^	43.51^a^	45.37^ab^	1.354	<0.001	<0.001	0.041
VW (μm)	101.73^c^	100.31^c^	93.59^b^	88.35^ab^	84.93^a^	85.64^a^	1.754	<0.001	<0.001	0.022
VH (μm)	351.00^c^	352.20^c^	327.83^c^	307.49^bc^	276.42^ab^	247.26^a^	10.437	0.001	<0.001	0.859
CD (μm)	64.32^c^	64.49^c^	64.20^c^	59.66^bc^	56.83^ab^	50.64^a^	1.448	0.005	<0.001	0.317
VH/CD	5.52^b^	5.53^b^	5.19^ab^	5.24^ab^	4.97^a^	4.89^a^	0.074	0.017	0.001	0.112

Different superscript letters within a row indicate significant differences (*P* < 0.05, *n* = 3 cage-level biological replicates). MT, muscularis thickness; VW, villus width; VH, villus height; CD, crypt depth; VH/CD, villus height-to-crypt depth ratio.

Scanning electron microscopy observations revealed that the distal intestinal microvilli of CN7.3 and CN8.1 groups were dense and displayed numerous secretory pores (Figure 1). In contrast, as dietary C:N ratios increased further, abnormal goblet cell secretion was observed, and the microvilli generally appeared atrophied and sparse.

**FIGURE 1:**
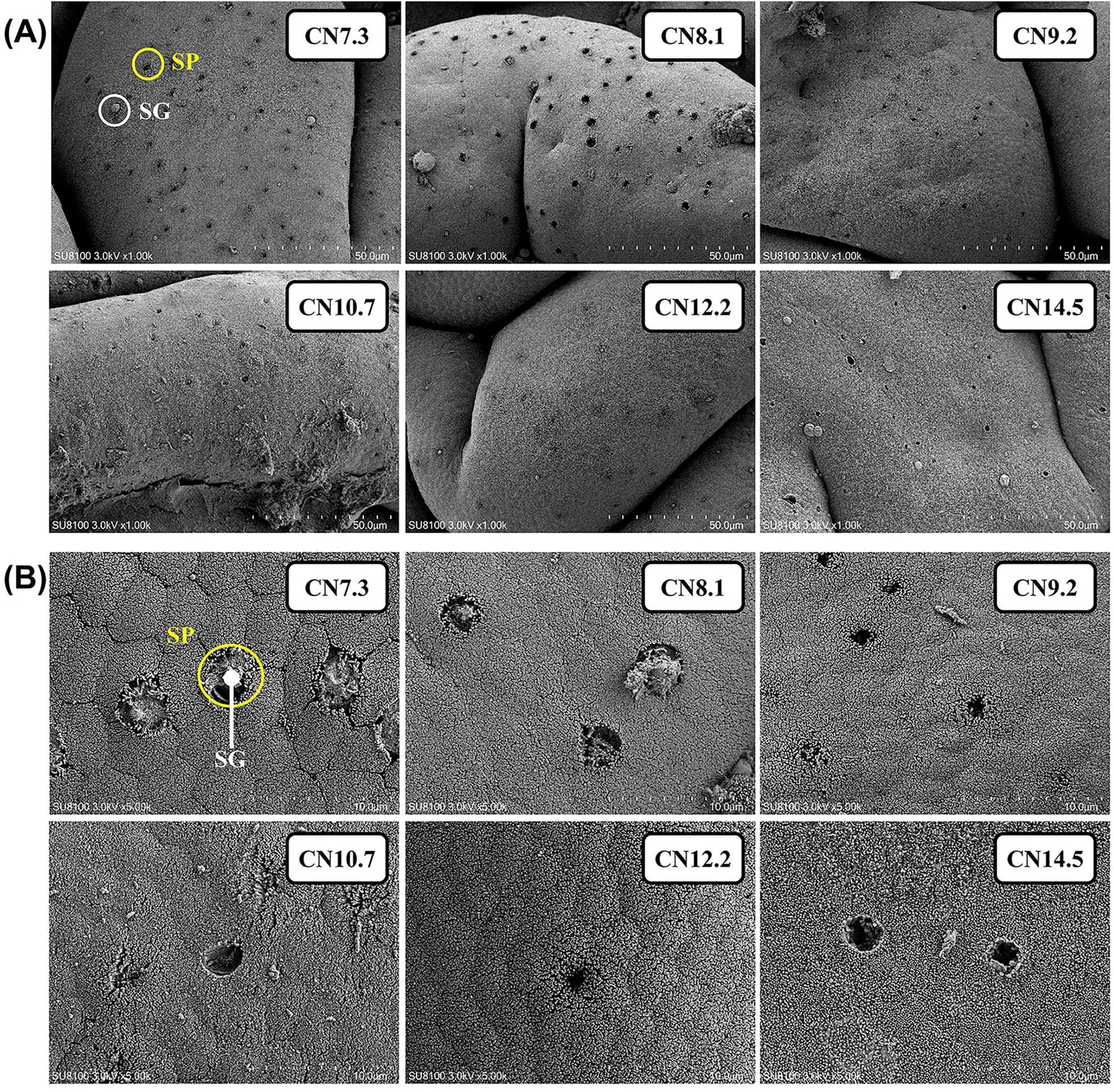
Effects of dietary CN ratios on distal intestinal morphology (scanning electron microscopy) of GIFT. **(A)** 1,000× magnification; **(B)** 5,000× magnification. SP, secretory pores; SG, secretory granules. CN7.3 and CN8.1 group exhibited dense microvilli with numerous SP. With increasing dietary CN ratios, abnormal goblet cell secretion was observed, and the microvilli appeared atrophied and sparse.

### 3.3 Intestinal digestive function

Trypsin activity generally decreased as dietary C:N ratios increased; activity in the CN7.3 and CN8.1 groups was significantly higher than CN10.7, CN12.2, and CN14.5 groups (*P* < 0.001, Table 5). In contrast, the highest AMS activity was observed in the CN12.2 group and was significantly higher than that in the CN7.3–CN10.7 groups. Additionally, trypsin activity and AMS activity showed significant linear trends across the ordered dietary treatments (*P* < 0.001).

**TABLE 5:** Effects of dietary C:N ratios on the proximal intestine digestive enzyme activities of GIFT.

Items	Groups						SEM	*P*-value		
	CN7.3	CN8.1	CN9.2	CN10.7	CN12.2	CN14.5		ANOVA	Linear	Quadratic
Trypsin (U/g protein)	676.41^d^	631.91^d^	602.79^c^	471.60^b^	365.31^ab^	349.65^a^	31.657	<0.001	<0.001	0.291
Lipase (U/g protein)	5.38	5.64	6.49	5.16	5.76	5.00	0.224	0.503	0.551	0.256
AMS (U/mg protein)	3.13^a^	3.65^a^	5.36^b^	5.33^b^	7.91^c^	6.58^bc^	0.433	<0.001	<0.001	0.247

Different superscript letters within a row indicate significant differences (*P* < 0.05, *n* = 3 cage-level biological replicates). AMS, α-amylase.

### 3.4 Intestinal mucosal barrier function

D-LA and LPS levels, together with DAO activity and ET levels, generally increased as dietary C:N ratios increased (*P* < 0.001; Table 6). Specifically, D-LA and LPS levels were significantly higher in the CN10.7–CN14.5 groups than in the CN7.3–CN9.2 groups. DAO activity generally increased in the higher-C:N treatments. ET levels also tended to increase, with the highest value observed in CN14.5; however, ET in CN10.7 was intermediate and did not differ significantly from the low-C:N groups. Additionally, all of D-LA, LPS, DAO and ET levels showed significant linear trends across the ordered dietary treatments (*P* < 0.001), whereas only ET level showed a significant quadratic trend (*P* < 0.001).

**TABLE 6:** Effects of dietary C:N ratios on plasma indices related to intestinal mucosal barrier function of GIFT.

Items	Groups						SEM	*P*-value		
	CN7.3	CN8.1	CN9.2	CN10.7	CN12.2	CN14.5		ANOVA	Linear	Quadratic
D-LA (μmol/L)	56.01^a^	57.51^a^	48.41^a^	76.28^b^	88.39^c^	80.91^bc^	3.778	<0.001	<0.001	0.265
DAO (U/mL)	16.63^b^	13.02^a^	12.09^a^	23.09^c^	21.50^c^	27.69^d^	1.932	0.008	<0.001	0.222
LPS (EU/L)	415.72^a^	506.29^b^	493.26^b^	686.56^c^	775.39^d^	746.49^cd^	34.304	<0.001	<0.001	0.383
ET (pg/mL)	67.92^a^	71.97^a^	65.70^a^	75.17^ab^	82.50^b^	83.03^b^	1.392	<0.001	<0.001	<0.001

Different superscript letters within a row indicate significant differences (*P* < 0.05, *n* = 3 cage-level biological replicates). D-LA, D-lactate; DAO, diamine oxidase; LPS, lipopolysaccharide; ET, endothelin.

### 3.5 Intestinal mucosal mechanical barrier

The mRNA expression of *zo-1*, *zo-3*, and *occludin* in the distal intestine generally decreased as dietary C:N ratio increased (*P* < 0.05, Figure 2A). Specifically, *zo-1* expression was significantly higher in the CN7.3 group than CN10.7–CN14.5 groups (*P* < 0.01), with the lowest level observed in CN12.2. For *zo-3*, the CN7.3 and CN9.2 groups exhibited significantly higher expression than the CN10.7–CN14.5 groups (*P* < 0.05). Similarly, *occludin* expression was significantly reduced in the CN14.5 group compared with the CN7.3–CN9.2 (*P* < 0.01). The expression of *claudin* showed a declining trend at higher C:N ratios, with CN14.5 showing significantly lower levels than CN7.3 and CN8.1 (*P* < 0.01). In addition, *claudin-12* expression was significantly elevated in CN8.1 compared with CN10.7, CN12.2 (*P* < 0.01). The expression levels of *zo-1*, *zo-3*, *occludin*, *claudin*, *claudin-11*, and *claudin-12* showed significant linear trends across the ordered dietary treatments (*P* < 0.05).

**FIGURE 2:**
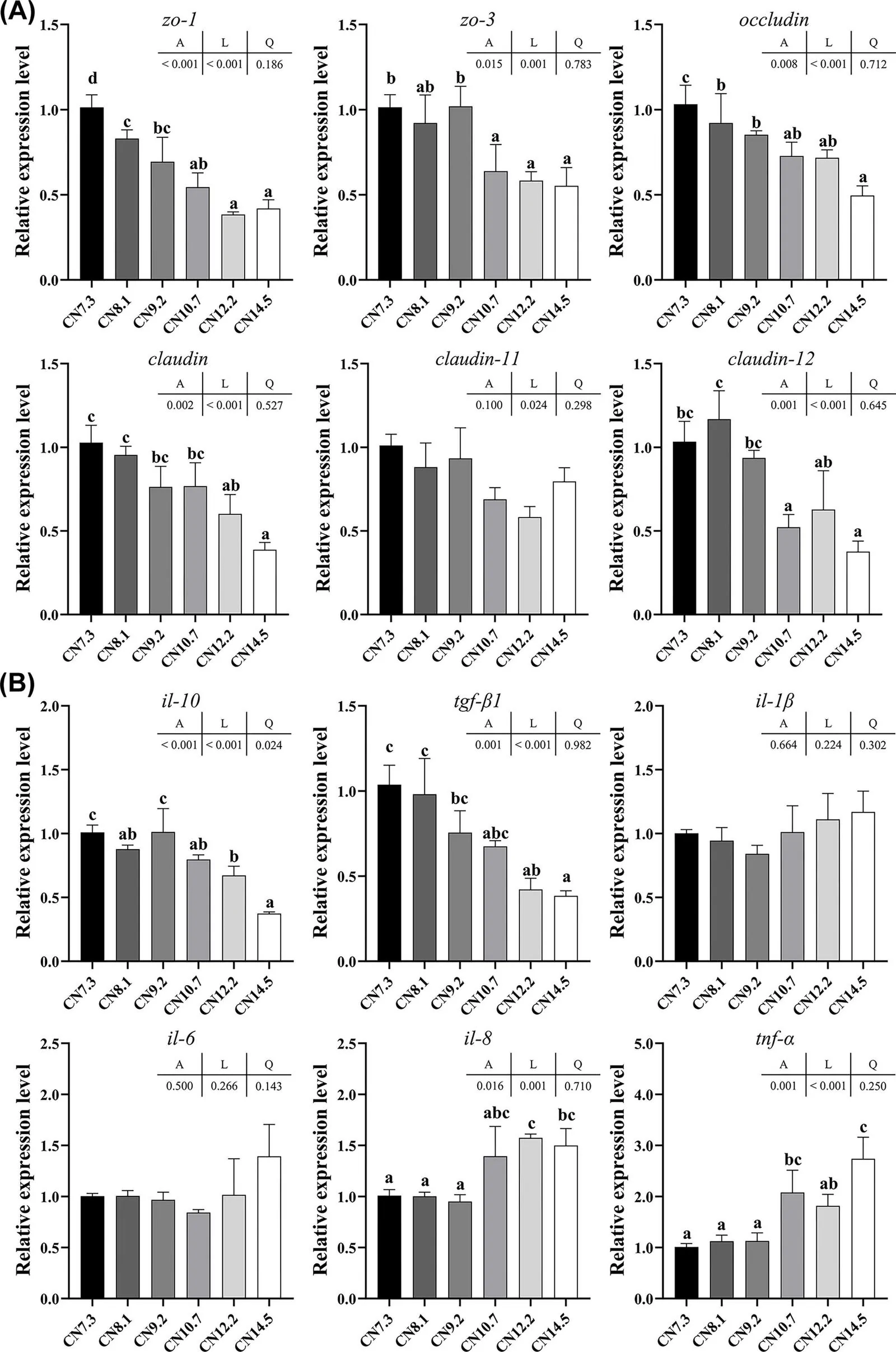
Effects of dietary CN ratios on the relative mRNA expression related to intestinal mucosal mechanical and immune barriers in the distal intestine of GIFT. **(A)** Genes related to the intestinal mucosal mechanical barrier; **(B)** genes related to the intestinal mucosal immune barrier. *zo-1*, zonula occludens 1; *zo-3*, zonula occludens 3; *tnf-α*, tumor necrosis factor α; *il-1β*, interleukin-1β; *il-6*, interleukin 6; *il-8*, interleukin 8; *il-10*, interleukin 10; *tgf-β1*, transforming growth factor β1. Values are expressed as means ± SEM (*n* = 3 cage-level biological replicates), bars with different superscript letters differ significantly (*P* < 0.05).

### 3.6 Intestinal mucosal immune barrier

Distal intestinal *il-10* expression was significantly higher in the CN7.3 and CN9.2 groups than CN12.2 and CN14.5 groups, with the lowest level observed in CN14.5 (*P* < 0.01, Figure 2B). For *tgf-β1*, the CN7.3 and CN8.1 groups showed significantly higher expression than CN12.2 and CN14.5 (*P* < 0.01). In contrast, *il-8* expression was significantly elevated in the CN12.2 and CN14.5 groups than CN7.3–CN9.2 (*P* < 0.05). Similarly, *tnf-α* expression was significantly higher in the CN10.7 and CN14.5 groups than in CN7.3–CN9.2 groups (*P* < 0.01). The expression levels of *il-10*, *tgf-β1*, *il-8*, and *tnf-α* showed significant linear trends across the ordered dietary treatments (*P* < 0.05); only expression level of *il-10* showed a significant quadratic trend (*P* = 0.024).

### 3.7 Intestinal mucosal antioxidant parameters

The activities of GSH-Px, T-AOC, T-SOD, and CAT generally decreased as dietary C:N ratio increased (*P* < 0.05, Table 7). The CN7.3 and CN8.1 groups exhibited higher antioxidant enzyme activities than the CN12.2 and CN14.5 groups, with GSH-Px, T-AOC, and T-SOD showing marked reductions at higher C:N ratios (*P* < 0.05). CAT activity showed a similar decline and was lowest in the CN14.5 group (*P* < 0.001). In contrast, contents of GSH and MDA did not show a consistent pattern (*P* > 0.05). Additionally, T-AOC, GSH-Px, T-SOD, and CAT levels showed significant linear trends across the ordered dietary treatments (*P* < 0.001).

**TABLE 7:** Effects of dietary C:N ratios on intestinal antioxidant capacity of GIFT.

Items	Groups						SEM	*P*-value		
	CN7.3	CN8.1	CN9.2	CN10.7	CN12.2	CN14.5		ANOVA	Linear	Quadratic
GSH (μmol/g protein)	69.62	66.73	54.38	61.30	61.92	53.25	3.092	0.656	0.205	0.759
GSH-Px (U/mg protein)	37.18^c^	37.08^c^	36.65^c^	34.91^bc^	32.98^ab^	31.87^a^	0.573	0.020	<0.001	0.164
T-AOC (mmol/g protein)	0.91^d^	0.94^cd^	0.75^bc^	0.80^c^	0.62^a^	0.58^a^	0.036	<0.001	< 0.001	0.390
T-SOD (U/mg protein)	89.75^d^	82.11^cd^	70.54^bc^	79.49^cd^	65.08^ab^	56.12^a^	3.122	0.018	<0.001	0.669
CAT (U/mg protein)	4.11^c^	3.69^bc^	3.64^bc^	3.44^bc^	3.14^b^	2.13^a^	0.167	<0.001	<0.001	0.057
MDA (nmol/mL)^1^	6.46	5.86	6.29	6.67	6.62	5.74	0.234	0.852	0.864	0.576

Different superscript letters within a row indicate significant differences (*P* < 0.05, *n* = 3 cage-level biological replicates). GSH, reduced glutathione; GSH-Px, glutathione peroxidase; T-AOC, total antioxidant capacity; T-SOD, total superoxide dismutase; MDA, malondialdehyde; CAT, catalase.

^1^For MDA determination, the protein concentration of the homogenate was adjusted to 3.5 mg/mL.

### 3.8 Intestinal microbiota

The sequencing depth after quality control was generally comparable among samples, with an average of 69,743.78 clean reads and an SEM of 555.41 (Figure 3A). These clean-read values represented the upstream quality-controlled sequencing output. After subsequent ASV processing and filtering, rarefaction curves were constructed using the final ASV count table to evaluate sequencing-depth sufficiency and inter-group differences in microbial richness. All rarefaction curves rose sharply at low sequencing amounts and gradually plateaued when the number of sampled sequences reached approximately 40,000–60,000, indicating that the sequencing depth was sufficient to capture the vast majority of ASVs in the samples (Figure 3B). In the final filtered ASV count matrix used for Bray–Curtis/PERMANOVA, the effective library sizes ranged from 50,983 to 67,180 counts per sample, with a mean of 61,094.5 ± 986.9 counts per sample (Supplementary Table 1 and Supplementary Data Sheet 1).

**FIGURE 3:**
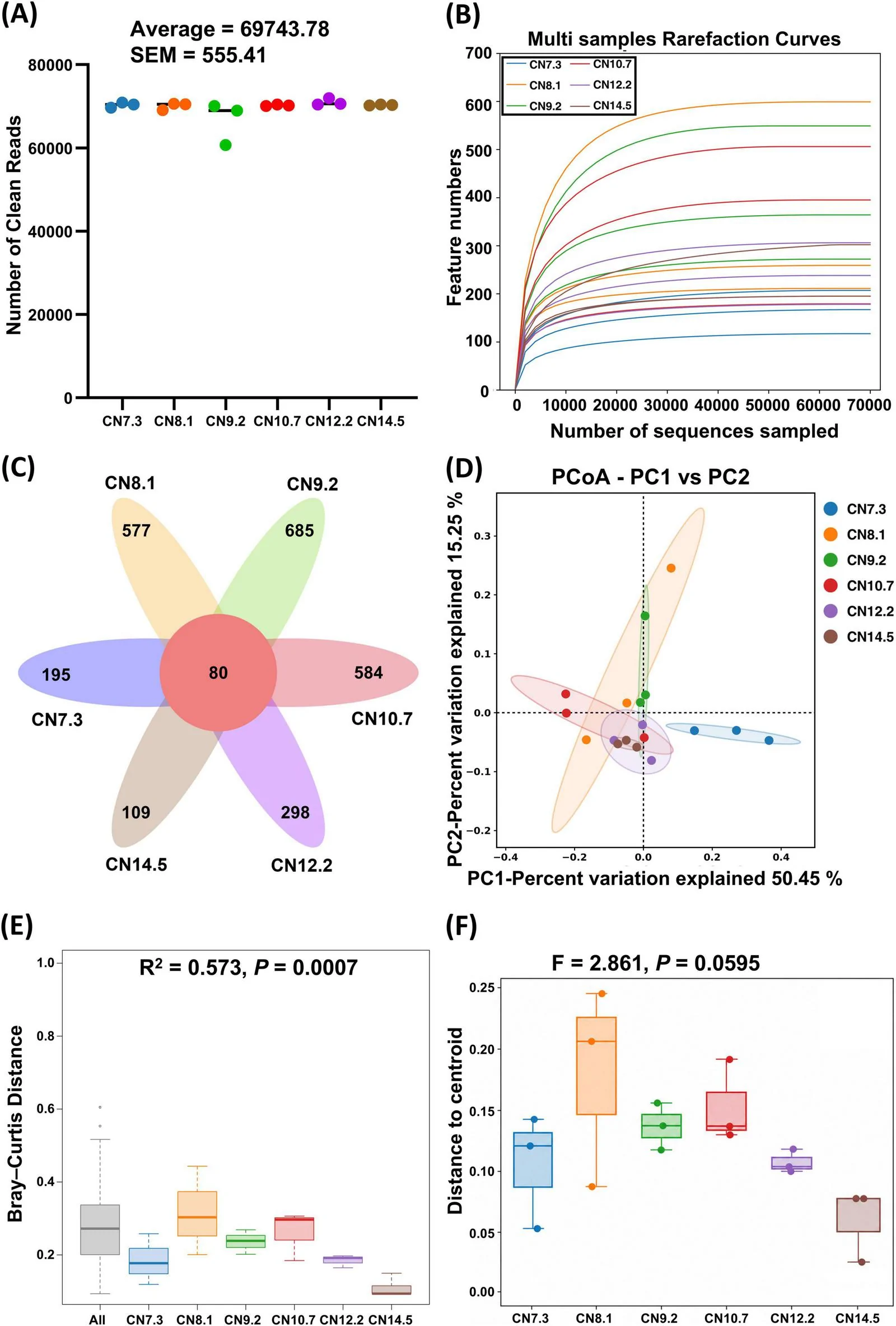
Effects of dietary CN ratios on microbiota numbers of clean read, rarefaction curves, ASV composition, and beta-diversity of GIFT. **(A)** Numbers of clean read; **(B)** Rarefaction curves based on ASVs; **(C)** Venn diagrams comparing the ASVs communities; **(D)** Principal coordinates analysis (PCoA) based on Bray–Curtis dissimilarities; **(E)** PERMANOVA based on Bray–Curtis distances; **(F)** Betadisper Analysis Based on Bray–Curtis Dissimilarities.

At the ASV level, the Venn diagram showed that 80 ASVs were shared across all six groups (Figure 3C). The CN9.2 group had the largest number of unique ASVs (685), whereas the CN14.5 group had the fewest (109). Principal coordinates analysis (PCoA) based on beta diversity showed a partial separation among groups. The first two principal coordinate axes explained 65.70% of the total variation, with PC1 and PC2 accounting for 50.45% and 15.25%, respectively (Figure 3D). PERMANOVA based on Bray–Curtis dissimilarities indicated significant differences in microbial community composition among groups (*R^2^* = 0.573, *P* = 0.0007, Figure 3E). This conclusion was robust to library-size handling: PERMANOVA after total-sum scaling to relative abundance yielded *R^2^* = 0.5713, pseudo-*F* = 3.198, and *P* = 0.0006, whereas PERMANOVA after rarefying all samples to 50,983 counts per sample yielded *R^2^* = 0.5717, pseudo-F = 3.203, and *P* = 0.0006 (Supplementary Table 2 and Supplementary Data Sheet 1). PERMDISP did not detect a significant difference in multivariate dispersion among groups (*F* = 2.861, *P* = 0.0595, Figure 3F).

Alpha diversity analysis showed significant among-treatment differences in Simpson and Shannon indices, whereas ACE and Good’s coverage did not differ significantly among treatments (Table 8). Simpson diversity was significantly lower in CN7.3 than in the other dietary groups (*P* < 0.001) and showed both significant linear and quadratic trends across the ordered treatments (*P* < 0.001). Shannon diversity was also lower in CN7.3 than in the remaining groups (*P* = 0.001) and showed significant linear (*P* = 0.022) and quadratic (*P* < 0.001) trends across the ordered treatments. ACE showed no significant overall or linear effect, although a significant quadratic trend was detected (*P* = 0.039).

**TABLE 8:** Effects of dietary C:N ratios on alpha indices of microbiota of GIFT.

Items	Groups						SEM	*P*-value		
	CN7.3	CN8.1	CN9.2	CN10.7	CN12.2	CN14.5		ANOVA	Linear	Quadratic
ACE	163.97	356.65	395.10	360.68	241.26	237.52	75.854	0.279	0.984	0.039
Simpson	0.48á	0.78á	0.74á	0.80á	0.75á	0.75á	0.035	<0.001	<0.001	<0.001
Shannon	2.15á	3.99á	3.87á	4.04á	3.57á	3.44á	0.244	0.001	0.022	<0.001
Good’s coverage	1.00	1.00	1.00	1.00	1.00	1.00	0.000	0.472	0.136	0.250

Different superscript letters within a row indicate significant differences (*P* < 0.05, *n* = 3 cage-level biological replicates). ACE: abundance-based coverage estimator.

The dominant phyla in the microbiota were Fusobacteriota, Firmicutes, Proteobacteria, Actinobacteriota, Bacteroidota, Chloroflexi, Cyanobacteria, Desulfobacterota, and Verrucomicrobiota (Figure 4A). The relative abundance of Fusobacteriota was highest in the CN7.3 group and showed significant differences among dietary treatments in the primary relative-abundance analysis (FDR-adjusted *P* = 0.005, Table 9). ANCOM-BC2 sensitivity analysis supported the overall treatment effect (Global *q* < 0.001), whereas neither the linear nor the quadratic trend detected in the primary analysis was retained after compositional adjustment (Supplementary Table 3 and Supplementary Data Sheet 2). Proteobacteria also differed significantly among dietary treatments (FDR-adjusted *P* = 0.047), with relative abundance higher in the CN8.1 and CN9.2 groups than in the CN7.3 and CN14.5 groups. A significant quadratic trend was detected across the ordered dietary treatments (FDR-adjusted *P* = 0.036). ANCOM-BC2 supported the overall treatment effect for Proteobacteria (Global *q* = 0.004), whereas the quadratic trend was not retained after compositional adjustment. Actinobacteriota increased significantly as dietary C:N ratio increased (FDR-adjusted *P* < 0.001). Additionally, Actinobacteriota showed a significant positive linear trend across the ordered dietary treatments (FDR-adjusted *P* < 0.001) which was retained in the ANCOM-BC2 sensitivity analysis (Linear *q* < 0.001).

**FIGURE 4:**
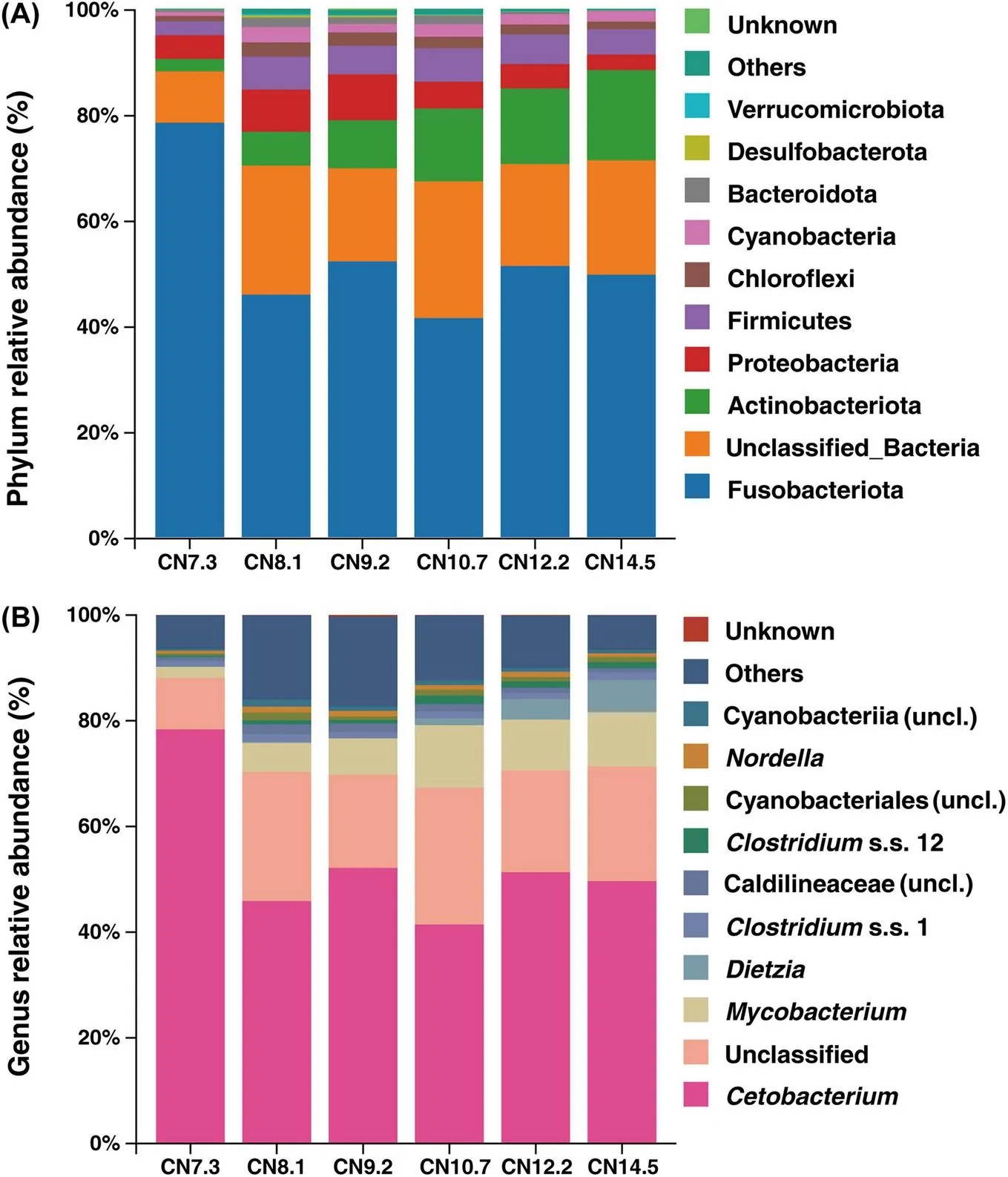
Effects of dietary CN ratios on relative abundance of microbiota of GIFT. **(A)** Stacked bar plot at the phylum level; **(B)** Stacked bar plot at the genus level. s.s., sensu stricto; uncl., unclassified.

**TABLE 9:** Effects of dietary C:N ratios on the relative abundance of phylum-level taxa and the top 10 genus-level taxa in the intestinal microbiota of GIFT.

Items	Groups						SEM	*P*-value			
	CN7.3	CN8.1	CN9.2	CN10.7	CN12.2	CN14.5		ANOVA	FDR- adjusted^1^ ANOVA	FDR- adjusted Linear	FDR- adjusted Quadratic
Phylum-level											
Fusobacteriota (%)	78.27^b^	45.82^a^	52.27^a^	41.12^a^	51.26^a^	49.66^a^	3.252	0.001	0.005	0.013	0.011
Firmicutes (%)	2.67	6.28	5.47	6.42	5.64	4.78	0.445	0.130	0.293	0.542	0.073
Proteobacteria (%)	4.51^a^	7.97^bc^	8.61^c^	5.08^ab^	4.64^ab^	2.93^a^	0.604	0.016	0.047	0.090	0.036
Actinobacteriota (%)	2.38^a^	6.40^b^	9.12^b^	13.77^c^	14.29^cd^	17.07^d^	1.272	<0.001	<0.001	<0.001	0.172
Bacteroidota (%)	0.36	1.76	1.30	1.73	0.47	0.10	0.324	0.568	0.568	0.565	0.172
Chloroflexi (%)	0.97	2.70	2.52	2.17	1.87	1.46	0.240	0.300	0.410	0.942	0.111
Cyanobacteria (%)	0.79	2.93	1.56	2.36	1.95	2.03	0.243	0.171	0.307	0.542	0.229
Desulfobacterota (%)	0.05	0.43	0.38	0.13	0.02	0.04	0.077	0.527	0.568	0.542	0.274
Verrucomicrobiota (%)	0.03	0.31	0.19	0.17	0.08	0.05	0.039	0.319	0.410	0.542	0.172
Genus-level											
*Cetobacterium* (%)	78.25^b^	45.82^a^	52.25^a^	41.11^a^	51.26^a^	49.65^a^	3.250	0.001	0.012	0.019	0.037
*Mycobacterium* (%)	2.10^a^	5.50^ab^	6.94^bc^	11.87^d^	9.60^bcd^	10.31^cd^	0.930	0.004	0.032	0.004	0.230
*Dietzia* (%)	0.02^a^	0.23^a^	0.09^a^	1.22^a^	3.88^b^	6.06^b^	0.605	<0.001	0.007	<0.001	0.080
*Clostridium* (s.s 1) (%)	1.13	1.40	1.16	1.28	1.12	1.49	0.114	0.940	0.940	0.839	0.789
Caldilineaceae (uncl.) (%)	0.70	1.86	1.50	1.39	1.06	0.74	0.169	0.310	0.586	0.729	0.230
*Clostridium* (s.s 12) (%)	0.30	0.82	0.69	1.59	1.16	1.17	0.126	0.025	0.122	0.037	0.230
*Nordella* (%)	0.49	1.16	1.06	0.89	1.02	0.70	0.106	0.507	0.598	0.879	0.230
Cyanobacteriales (uncl.) (%)	0.39	1.42	0.66	1.15	0.82	0.97	0.124	0.200	0.444	0.729	0.360
Cyanobacteriia (uncl.) (%)	0.37	1.31	0.75	0.89	0.73	0.65	0.112	0.269	0.539	0.918	0.252
*Methylocystis* (%)	0.23	0.64	0.68	0.63	0.59	0.60	0.064	0.382	0.598	0.482	0.230

Different superscript letters within a row indicate significant differences (*P* < 0.05, *n* = 3 cage-level biological replicates). Statistical tests were performed directly on taxon relative-abundance data without log-ratio or other compositional transformation. Statistical results in this table represent the primary relative-abundance analysis. Compositional robustness of taxonomic findings was further evaluated using ANCOM-BC2 sensitivity analysis (Supplementary Table 3 and Supplementary Data Sheet 2).

^1^For multiple-testing correction, features with a mean relative abundance > 0.1% were included, and *P*-values were adjusted using the Benjamini–Hochberg false discovery rate (FDR) method.

Genus-level analysis (Figure 4B) revealed a community dominated by *Cetobacterium*, *Mycobacterium*, *Dietzia*, *Clostridium* sensu stricto 1, *Clostridium s*ensu stricto 12, and *Nordella*. The relative abundance of *Cetobacterium* was highest in the CN7.3 group compared with the other dietary groups (FDR-adjusted *P* = 0.012, Table 9), consistent with ANCOM-BC2, which identified a significant overall treatment effect for this genus (Global *q* < 0.001). The relative abundance of *Mycobacterium* in the CN9.2–CN14.5 groups was significantly higher than CN7.3 group, with the highest values observed in CN10.7 (FDR-adjusted *P* = 0.032); ANCOM-BC2 also supported an overall treatment effect (Global *q* < 0.001). The relative abundance of *Dietzia* in the CN12.2 and CN14.5 groups was significantly higher than that in the other groups (FDR-adjusted *P* = 0.007), and the overall treatment effect was supported by ANCOM-BC2 (Global *q* < 0.001). Additionally, *Mycobacterium*, *Dietzia*, and *Clostridium sensu* stricto 12 showed significant linear trends across the ordered dietary treatments (FDR-adjusted *P* < 0.05), and were supported by ANCOM-BC2 sensitivity analysis (*q* < 0.05).

Functional prediction using PICRUSt2 revealed significant differences among several of the top 10 KEGG pathways with relative abundance (Table 10). In the CN7.3 group, predicted gene abundances associated with “Biosynthesis of secondary metabolites,” “Biosynthesis of antibiotics” and “Microbial metabolism” were significantly lower than those in the other groups (FDR-adjusted *P* < 0.05). Conversely, predicted gene abundances related to “Ribosome,” “Purine metabolism,” and “Quorum sensing” in CN7.3 group were significantly higher than those in the other groups (FDR-adjusted *P* < 0.01). In addition, “ABC transporters” were higher in the CN7.3, CN8.1, and CN9.2 groups, all relative to the CN14.5 group (FDR-adjusted *P* = 0.003). Additionally, “Metabolic pathways,” “Biosynthesis of secondary metabolites,” “Biosynthesis of antibiotics,” “Microbial metabolism,” “ABC transporters,” “Ribosome,” “Purine metabolism,” and “Quorum sensing” all showed significant linear trends across the ordered dietary treatments (FDR-adjusted *P* < 0.05). Significant quadratic trends were also detected for “Microbial metabolism,” “Ribosome,” and “Purine metabolism” (FDR-adjusted *P* < 0.05).

**TABLE 10:** Effects of dietary C:N ratios on top 10 predicted functional profiles of microbiota of GIFT using PICRUSt2 and FAPROTAX.

Items	Groups						SEM	*P*-value			
	CN7.3	CN8.1	CN9.2	CN10.7	CN12.2	CN14.5		ANOVA	FDR- adjusted^1^ ANOVA	FDR- adjusted Linear	FDR- adjusted Quadratic^2^
PICRUSt2 with KEGG											
Metabolic pathways (%)	17.22	17.13	16.95	16.94	16.93	16.85	0.040	0.035	0.053	0.003	0.368
Biosynthesis of secondary metabolites (%)	7.23^a^	7.47^b^	7.39^b^	7.51^b^	7.44^b^	7.49^b^	0.028	0.019	0.030	0.012	0.119
Biosynthesis of antibiotics (%)	5.09^a^	5.23^b^	5.23^b^	5.27^b^	5.25^b^	5.31^b^	0.020	0.004	0.008	<0.001	0.133
Microbial metabolism (%)	4.05^a^	4.20^b^	4.29^bc^	4.37^c^	4.34^c^	4.35^c^	0.030	<0.001	0.002	<0.001	0.042
ABC transporters (%)	3.91^d^	3.68^bc^	3.72^c^	3.54^ab^	3.58^abc^	3.46^a^	0.038	<0.001	0.003	<0.001	0.230
Amino acids synthesis (%)	3.14	3.20	3.17	3.15	3.12	3.20	0.016	0.708	0.720	0.959	0.824
Carbon metabolism (%)	2.63	2.62	2.65	2.63	2.65	2.65	0.035	0.309	0.348	0.102	0.833
Ribosome (%)	2.34^b^	2.14^a^	2.13^a^	2.02^a^	2.08^a^	2.06^a^	0.028	0.001	0.004	<0.001	0.042
Purine metabolism (%)	2.22^b^	2.04^a^	2.04^a^	1.97^a^	2.03^a^	2.00^a^	0.022	0.002	0.006	0.002	0.042
Quorum sensing (%)	1.91^d^	1.69^bc^	1.74^c^	1.58^ab^	1.61^abc^	1.53^a^	0.032	<0.001	0.002	<0.001	0.095
FAPROTAX											
Chemoheterotrophy (%)	32.32^b^	30.41^a^	31.33^ab^	31.73^b^	32.35^b^	32.32^b^	0.212	0.016	0.049	0.143	0.078
Fermentation (%)	30.92^b^	25.84^a^	26.33^a^	23.46^a^	24.59^a^	24.10^a^	0.677	0.001	0.007	0.001	<0.050
Nitrate reduction (%)	30.29^c^	24.12^ab^	25.26^b^	21.53^a^	23.68^ab^	23.10^ab^	0.755	0.001	0.007	0.002	0.046
Aerobic chemoheterotrophy (%)	1.31^a^	4.24^b^	4.75^b^	7.91^c^	7.41^c^	7.95^c^	0.616	<0.001	<0.001	<0.001	0.046
Ureolysis (%)	0.23^a^	0.54^ab^	0.69^ab^	1.14^b^	2.07^c^	2.99^d^	0.251	<0.001	0.001	<0.001	0.074
Hydrocarbon degradation (%)	0.24^a^	1.10^b^	1.07^b^	1.75^b^	1.36^b^	1.33^b^	0.134	0.008	0.029	0.010	<0.050
Phototrophy (%)	0.37	1.73	0.97	1.36	1.00	1.00	0.152	0.178	0.273	0.717	0.159
Photoautotrophy (%)	0.31	1.57	0.74	1.27	0.89	0.93	0.145	0.171	0.273	0.717	0.207
Aromatic compound degradation (%)	0.13^a^	0.65^b^	0.73^b^	1.35^c^	1.06^bc^	1.05^bc^	0.106	0.001	0.007	<0.001	0.046
Parasites or symbionts (%)	0.65^ab^	0.91^b^	0.90^b^	0.64^ab^	0.29^a^	0.32^a^	0.073	0.011	0.038	0.012	0.082

Different superscript letters within a row indicate significant differences (*P* < 0.05, *n* = 3 cage-level biological replicates). Predicted functional profiles are presented as relative abundances (%), and statistical analyses were performed directly on the untransformed relative-abundance values.

^1^For multiple-testing correction, features with a mean relative abundance > 0.1% were included, and *P*-values were adjusted using the Benjamini–Hochberg false discovery rate (FDR) method.

^2^Values reported as <0.050 indicate unrounded FDR-adjusted *P*-values below 0.050 that round to 0.050 when expressed to three decimal places. PICRUSt2, Phylogenetic Investigation of Communities by Reconstruction of Unobserved States 2; FAPROTAX, Functional Annotation of Prokaryotic Taxa; KEGG, Kyoto Encyclopedia of Genes and Genomes; ABC transporters, adenosine triphos phate binding cassette.

Functional Annotation of Prokaryotic Taxa functional prediction indicated that the chemoheterotrophy function in the CN8.1 group was significantly lower than CN7.3, CN10.7, and CN14.5 groups (FDR-adjusted *P* = 0.049, Table 10). Fermentation and nitrate reduction were significantly enriched in the CN7.3 group relative to all other groups (FDR-adjusted *P* = 0.007). In contrast, aerobic chemoheterotrophy and hydrocarbon degradation were reduced in CN7.3 compared with the other groups, whereas ureolysis was lower in CN7.3 than in the higher C:N ratio groups (FDR-adjusted *P* < 0.05). Aromatic compound degradation was significantly reduced in the CN7.3 group compared with all other groups, with the highest value observed in the CN10.7 group (FDR-adjusted *P* = 0.007). Parasites or symbionts were significantly enriched in the CN8.1 and CN9.2 groups compared with the CN12.2 and CN14.5 groups, whereas CN7.3 and CN10.7 showed intermediate values (FDR-adjusted *P* = 0.038). Additionally, fermentation, nitrate reduction, aerobic chemoheterotrophy, ureolysis, hydrocarbon degradation, aromatic compound degradation, and parasites or symbionts exhibited significant linear trends across the ordered dietary treatments (FDR-adjusted *P* < 0.05), while nitrate reduction, fermentation, aerobic chemoheterotrophy, hydrocarbon degradation, and aromatic compound degradation also showed significant quadratic trends (FDR-adjusted *P* < 0.05). Detailed supporting materials for these intestinal microbiota analyses, including underlying data and analytical procedures, are provided in Supplementary Data Sheet 3.

### 3.9 SCFA concentrations in intestinal contents

Acetic acid content was higher in the CN7.3 group than in all other groups (*P* < 0.01, Table 11), whereas no significant differences were observed among the CN8.1–CN14.5 groups. Propionic acid content fluctuated across treatments, with the highest level observed in the CN12.2 group, which was significantly higher than CN10.7 group and CN8.1 group (*P* < 0.05). Butyric acid content differed significantly among treatments (*P* < 0.05). The CN9.2, CN10.7, and CN14.5 groups had significantly higher butyric acid concentrations than the CN7.3 group, whereas CN8.1 and CN12.2 showed intermediate values. The highest numerical value was observed in CN10.7. In contrast, concentrations of valeric acid and caproic acid did not differ among groups (*P* > 0.05). The highest total SCFAs (ΣSCFA) level was observed in the CN7.3 group, which was significantly higher than other groups. The lowest value was recorded in the CN14.5 group (*P* < 0.01). Additionally, acetic acid and total SCFAs showed significant linear trends across the ordered dietary treatments (*P* < 0.01). Meanwhile, acetic acid and butyric acid exhibited significant quadratic trends across the ordered dietary treatments (*P* < 0.05).

**TABLE 11:** Effects of dietary C:N ratios on SCFA concentrations in intestinal contents (μg/g) of GIFT.

Items	Groups						SEM	*P*-value		
	CN7.3	CN8.1	CN9.2	CN10.7	CN12.2	CN14.5		ANOVA	Linear	Quadra
Acetic acid	38.96^b^	23.09^a^	24.43^a^	18.94^a^	27.15^a^	19.60^a^	1.886	0.003	0.002	0.020
Propionic acid	0.58^bc^	0.40^ab^	0.60^bc^	0.34^a^	0.74^c^	0.55^bc^	0.039	0.010	0.290	0.228
Butyric acid	0.80^a^	1.17^ab^	1.28^b^	1.60^b^	1.14^ab^	1.33^b^	0.078	0.044	0.069	0.028
Valeric acid	2.20	2.02	2.85	2.32	2.66	2.65	0.151	0.656	0.286	0.764
Caproic acid	7.56	7.37	8.70	6.74	7.94	4.43	0.489	0.166	0.100	0.104
ΣSCFA	50.09^d^	34.04^abc^	37.85^bc^	29.94^ab^	39.63^c^	28.55^a^	1.958	0.001	<0.001	0.067

Different superscript letters within a row indicate significant differences (*P* < 0.05, *n* = 3 cage-level biological replicates).

## 4 Discussion

In this study, the graded dietary C:N ratio treatments were generated by coordinated changes in dietary formulation, decreasing protein levels and increasing wheat starch and wheat bran. Therefore, dietary C:N ratio was not isolated as an independent variable, and the observed responses should be interpreted as the combined effects of dietary C:N ratio, crude protein level, and carbohydrate level under the present formulation strategy. Because fish generally exhibit limited capacity for efficient carbohydrate utilization (Wilson, 1994), the increased non-protein energy supplied in high C:N diets may not have been effectively converted into growth. Although feeding rate increased in the high C:N groups, the reduced final body weight and elevated FCR indicate that higher intake did not translate into improved feed utilization, likely reflecting compensatory feeding under reduced protein supply. Differences from previous tilapia studies (Liu et al., 2025) reporting improved performance under moderate protein-to-carbohydrate ratios (e.g., 27:35 vs. 35:25) may be attributable to variations in culture conditions, ingredient composition, or feeding management. Accordingly, the following discussion interprets the effects of graded dietary C:N ratio treatments within the context of this specific dietary formulation strategy rather than as responses to dietary C:N ratio alone.

Intestinal morphology is closely linked to nutrient digestion and absorption. Villus length, crypt depth, and microvilli structure are widely used indicators of intestinal absorptive capacity (Su et al., 2022), and longer villi with shallower crypts generally indicate increased mucosal surface area and improved absorption (Zaki et al., 2025). In this study, high C:N diets were associated with reduced mucosal surface characteristics and microvilli atrophy, suggesting impaired absorptive function, potentially due to insufficient protein/amino acid supply for intestinal development (Özel et al., 2018). Digestive enzyme profiles further supported diet-driven adaptation: trypsin activity decreased while α-amylase activity increased in the high C:N groups, consistent with observations in *Rachycentron canadum* (Wang et al., 2022) and *Micropterus salmoides* (Huang et al., 2021). This pattern suggests reduced demand for protein digestion alongside a compensatory increase in carbohydrate digestion capacity under carbohydrate-enriched diets.

The intestine is also a major immune and barrier organ (Deng et al., 2023). Plasma DAO activity and levels of LPS, ET, and D-LA are commonly used indicators of intestinal permeability and epithelial barrier damage (Deng et al., 2021). Elevated DAO reflects epithelial injury, whereas increased LPS and D-LA suggest barrier disruption with translocation of microbial components/metabolites, which can promote systemic inflammation (Lou et al., 2025). In this study, high C:N ratio groups (CN10.7–CN14.5) showed unfavorable changes in these plasma indicators, indicating compromised intestinal barrier integrity under excessive dietary C:N ratio. Consistent with these changes in permeability-related indicators, high C:N diets also downregulated several tight junction-related genes. Tight junction (TJ) proteins such as occludin, claudin, and zo-1 maintain epithelial integrity and regulate paracellular permeability (Han et al., 2023). In this study, high C:N diets downregulated barrier-related genes, suggesting weakened TJ architecture relative to low C:N groups. This impairment may be associated with reduced dietary protein/amino acids for epithelial maintenance and repair, and may also be exacerbated by shifts toward potentially opportunistic taxa. Consistently, bacterial infection can suppress TJ gene expression while reshaping intestinal microbial communities in cultured fish (Guo et al., 2024).

Antioxidant defense is essential for maintaining intestinal redox homeostasis. Enzymes such as GSH-Px, SOD, and CAT constitute primary antioxidant systems (Zhang et al., 2019). MDA is a key marker of lipid peroxidation (Liu et al., 2011), and GSH supports membrane protection and redox buffering (Abd El Hamid et al., 2024). In this study, reduced intestinal antioxidant capacity was most evident in the high C:N groups, indicating elevated oxidative stress. This may be linked to increased metabolic burden from carbohydrate-enriched diets and reduced amino acid availability for antioxidant enzyme synthesis. Barrier disruption and microbiota alterations may further contribute to oxidative and inflammatory processes in these treatments. Notably, MDA content did not show a significant change across treatments, despite the clear reductions in antioxidant enzyme activities. This may suggest that the observed oxidative stress was primarily at an early or compensatory stage, in which enzymatic defenses were depressed but overt lipid peroxidation had not yet accumulated to a detectable level. It is also plausible that non-enzymatic antioxidants such as GSH provided partial protection against membrane damage. These oxidative changes were accompanied by alterations in inflammatory regulation. Pro-inflammatory cytokines (IL-1β, IL-6, IL-8, TNF-α) and anti-inflammatory cytokines (IL-10, TGF-β1) jointly regulate inflammation and tissue repair (Sakai et al., 2021), and their balance is widely utilized to determine intestinal inflammatory status (Ingerslev et al., 2010). In this study, low C:N groups (CN7.3–CN9.2) exhibited relatively lower inflammatory status. In contrast, high C:N diets significantly downregulated *il-10* and *tgf-β1* and upregulated *il-8* and *tnf-α*, indicating induction of intestinal inflammation.

Dietary C:N ratio also markedly influenced intestinal microbiota structure. Intestinal microbiota can be classified as autochthonous (epithelium-associated) and allochthonous (digesta-associated) communities (Nyholm et al., 2022; Ruiz et al., 2024), and digesta composition can still reflect intestinal ecological status and functional tendencies (Soh et al., 2024). CN7.3 exhibited a comparatively simpler community structure, whereas higher C:N groups showed increased Shannon and Simpson. However, the increase in diversity observed in the higher C:N groups should be interpreted as microbial restructuring under altered nutrient supply rather than direct evidence of improved intestinal status, because it does not necessarily indicate improved intestinal health.

The dominant phyla (Fusobacteriota, Actinobacteriota, Firmicutes, Proteobacteria, Chloroflexi, Cyanobacteria, Bacteroidota, Desulfobacterota, Verrucomicrobiota) were consistent with previous reports (Huang et al., 2025). Fusobacteriota abundance showed a similar pattern to *Cetobacterium*, a core freshwater fish taxon linked to vitamin B_12_ production and acetate-generating fermentation (Larsen et al., 2014; Qi et al., 2023). The reduction in *Cetobacterium* occurred alongside broader diet-driven community restructuring, and ANCOM-BC2 supported an overall treatment effect after compositional adjustment, although a specific response pattern across treatments was not supported. Proteobacteria, often considered responsive to increased resource availability and copiotrophic conditions (Fierer et al., 2007; Osburn et al., 2025), increased in CN8.1 and CN9.2, but this increase was not accompanied by evident intestinal impairment, indicating that its abundance did not track intestinal impairment across treatments. Actinobacteriota, which include taxa capable of producing carbohydrate-active enzymes and utilizing polysaccharides (Kafilzadeh and Dehdari, 2015), also increased with higher dietary C:N ratio, consistent with previous observations (Qi et al., 2022), and this shift may be associated with the enhanced intestinal AMS activity observed in the high C:N groups. Overall, these changes suggest diet-associated restructuring of the intestinal microbiota and potential coordination with host digestive physiology, consistent with previous evidence linking fish intestinal microbial composition with digestive enzyme activity and nutrient metabolism (Luan et al., 2023).

At the genus level, *Dietzia* showed increased relative abundance in the higher C:N groups, and this pattern was supported by ANCOM-BC2 linear trend analysis. Given its environmental distribution and metabolic versatility (Bihari et al., 2011), this enrichment likely reflects diet-driven restructuring rather than direct evidence of intestinal disease, as fish-pathogenic evidence remains limited despite human clinical case reports (Bemer-Melchior et al., 1999; Brown et al., 2022). *Mycobacterium* showed both a significant overall treatment effect and a significant positive linear trend with increasing dietary C:N ratio in the primary analysis, and both signals were supported by ANCOM-BC2. This increasing trend warrants attention because some fish-associated mycobacteria can cause disease in susceptible hosts; however, genus-level 16S rRNA gene identification cannot confirm pathogenic species or active infection (Hashish et al., 2018), and these findings indicate enrichment of *Mycobacterium* rather than established disease risk. *Clostridium* sensu stricto 12 exhibited a significant positive linear trend with increasing dietary C:N ratios. Its mean relative abundance was numerically highest at CN10.7. This genus has been identified as a major butyrate-producing taxon involved in the metabolic conversion of lactate and acetate into butyrate (Detman et al., 2021), suggesting a possible association between its higher abundance and the elevated butyrate levels observed in this study. Together, these results indicate microbiota restructuring under increased dietary C:N ratio. A limitation of the taxon-level analysis is that relative abundances were evaluated using conventional univariate statistical tests without a log-ratio or other compositional transformation; therefore, these findings should be interpreted as differences in relative community composition rather than changes in absolute microbial abundance.

Functional prediction suggested that dietary C:N ratio altered the inferred metabolic potential of the microbiota. The CN7.3 showed higher fermentation and nitrate reduction functions, consistent with predominance of anaerobic intestinal processes central to microbial fermentation and nitrogen transformation (Dashora et al., 2025). With increasing C:N ratio, aerobic chemoheterotrophy was progressively enriched, consistent with higher carbon substrate availability favoring chemoheterotrophic potential. Ureolysis also increased under higher C:N, suggesting enhanced urea hydrolysis and nitrogen recycling within the microbiota (Shi et al., 2025). Under nitrogen-limited conditions, intestinal microbes can recover nitrogen from urea and other nitrogenous compounds to sustain growth, representing an important nitrogen recycling pathway in the gut ecosystem. Such nitrogen recycling strategies are widely recognized as microbial adaptations to nutrient imbalance and nitrogen limitation (Louca et al., 2016). Predicted hydrocarbon degradation functions increased with dietary C:N ratio, likely reflecting broader oxidative and complex carbon utilization assignments by FAPROTAX rather than indicating actual hydrocarbon exposure or true hydrocarbon degradation in the fish intestine (Fuirst et al., 2021). Such *in silico* predictions should be treated as tentative and require metagenomic or functional validation to clarify their biological relevance. These predictions represent inferred community potential and should be interpreted cautiously. More broadly, the metabolic potential of fish intestinal microbiota has been shown to influence host energy metabolism and nutrient utilization, highlighting the importance of microbial functional shifts in response to dietary manipulation (Butt and Volkoff, 2019).

Short-chain fatty acids produced by intestinal microbes are key mediators of host–microbiota interactions, contributing to epithelial energy supply, immune regulation, and intestinal barrier integrity in fish (Talwar et al., 2018). Moreover, these fermentation products can influence host metabolism and nutrient absorption. SCFA profiles varied among diets and were consistent with taxonomic shifts. Acetate was highest in CN7.3, aligning with *Cetobacterium* dominance (Wang et al., 2021). Butyrate was significantly higher in CN9.2, CN10.7, and CN14.5 than in CN7.3, whereas CN8.1 and CN12.2 showed intermediate values; the highest numerical butyrate concentration was observed in CN10.7. This numerical maximum coincided with the numerically highest mean relative abundance of *Clostridium sensu stricto* 12 at CN10.7, which, as noted above, is a characterized butyrate-producing taxon (Detman et al., 2021; Louis and Flint, 2017). SCFAs support mucosal barrier integrity and immune homeostasis and can enhance epithelial barrier function and reduce inflammation in fish (Shin et al., 2023). Notably, although butyrate was elevated in the CN14.5 groups, total SCFAs and acetate were highest in CN7.3, and the higher C:N treatments consistently showed poorer growth, intestinal morphology, barrier markers, antioxidant status, and inflammatory status. Thus, the butyrate increase should not be interpreted alone as evidence of improved intestinal condition; rather, the overall host response indicates that high C:N treatments did not necessarily improve intestinal health. Therefore, lower total SCFA levels in the higher C:N groups may partly contribute to the impaired intestinal morphology observed in this study. The present results indicate diet-dependent shifts in fermentation end-products, reflecting microbial metabolic adaptation to macronutrient composition; however, SCFA composition alone may not indicate an optimal intestinal state and should be considered together with host growth and intestinal health indicators.

Overall, under the present dietary formulation strategy, higher C:N ratios were associated with marked changes in intestinal microbiota structure and inferred functional potential. However, diets with C:N ratios ≥ 10.7 were associated with poorer growth efficiency, impaired intestinal morphology, increased permeability indicators, downregulation of barrier-related genes, reduced antioxidant capacity, and elevated inflammatory responses, whereas diets with C:N ratios up to 9.2 produced less pronounced adverse host responses under the present experimental conditions and formulation.

## 5 Conclusion

This study established distinct intestinal microbiota profiles in GIFT by feeding diets with graded C:N ratios (7.3–14.5). Alpha diversity differed among treatments, mainly reflecting lower Shannon and Simpson diversity in the CN7.3 group. Under high C:N conditions, the relative abundance of Actinobacteriota increased, including a marked increase in *Mycobacterium*. In parallel, butyrate differed among diets and was numerically highest in CN10.7, although this did not correspond to an improvement in host intestinal health. Functional prediction further indicated shifts in inferred microbial metabolic potential across dietary treatments. Collectively, these results indicate that the graded diet formulations were associated with shifts in intestinal microbial composition, including increased *Mycobacterium*, and changes in inferred carbohydrate-associated functional potential.

However, diets with C:N ratios ≥ 10.7 were associated with more pronounced adverse responses in growth performance, feed utilization efficiency, intestinal morphology, digestive enzyme activity, permeability indicators, inflammatory responses, and antioxidant capacity. Under the present formulation strategy, diets with C:N ratios up to 9.2 produced less pronounced adverse host responses than diets with ratios of 10.7 or higher. The present design did not identify a statistically optimized dietary C:N ratio or isolate the independent effect of C:N ratio from the concurrent changes in protein, carbohydrate, wheat bran, and ingredient composition.

## Data Availability

The datasets presented in this study can be found in online repositories. The names of the repository/repositories and accession number(s) can be found in the article/Supplementary material.

## Footnotes

1
https://cutadapt.readthedocs.io/

2
https://benjjneb.github.io/dada2

3
https://qiime2.org

4
http://www.arb-silva.de

## References

[B1] Abd El Hamid M. I. El-Azzouny M. M. El-Malt R. M. Elkenawy M. E. Abdelwarith A. A. Younis E. M. et al. (2024). Future impact of thymoquinone-loaded nanoemulsion in rabbits: Prospects for enhancing growth, immunity, antioxidant potential and resistance against *Pasteurella multocida*. *Front. Vet. Sci.* 10:1340964. 10.3389/fvets.2023.1340964 38292130 PMC10824920

[B2] AOAC (1990). *Official Methods of Analysis*, 15th Edn. Arlington, VA: Association of Official Analytical Chemists.

[B3] Basto-Silva C. García-Meilán I. Couto A. Serra C. R. Enes P. Oliva-Teles A. et al. (2022). Effect of dietary plant feedstuffs and protein/carbohydrate ratio on gilthead seabream (*Sparus aurata*) gut health and functionality. *Fishes* 7:59. 10.3390/fishes7020059

[B4] Bemer-Melchior P. Haloun A. Riegel P. Drugeon H. (1999). Bacteremia due to *Dietzia maris* in an immunocompromised patient. *Clin. Infect. Dis.* 29, 1338–1340. 10.1086/313490 10524995

[B5] Bihari Z. Szvetnik A. Szabó Z. Blastyák A. Zombori Z. Balázs M. et al. (2011). Functional analysis of long-chain n-alkane degradation by *Dietzia spp*. *FEMS Microbiol. Lett.* 316, 100–107. 10.1111/j.1574-6968.2010.02198.x 21204932

[B6] Brown W. D. Feinberg N. Stedman E. Dejace J. Hale A. J. (2022). *Dietzia cinnamea: An increasingly recognized human pathogen. IDCases* 29:e01539. 10.1016/j.idcr.2022.e01539 35756699 PMC9218238

[B7] Butt R. L. Volkoff H. (2019). Gut microbiota and energy homeostasis in fish. *Front. Endocrinol.* 10:9. 10.3389/fendo.2019.00009 30733706 PMC6353785

[B8] Chen J. Feng J. Zhu J. Luo L. Lin S. Wang D. et al. (2020). Starch to protein ratios in practical diets for genetically improved farmed Nile tilapia *Oreochromis niloticus*: Effects on growth, body composition, peripheral glucose metabolism and glucose tolerance. *Aquaculture* 515:734538. 10.1016/j.aquaculture.2019.734538

[B9] Dashora M. Kumar M. Kaushik G. Kumar A. (2025). Exploring biogeochemical transformation in a hypertrophic lake with dual focus on environmental cues and potential functional fingerprints of littoral bacteria. *Sci. Rep.* 15:31115. 10.1038/s41598-025-16527-y 40851025 PMC12375747

[B10] Dauda A. B. Romano N. Ebrahimi M. Teh J. C. Ajadi A. Chong C. M. et al. (2018). Influence of carbon/nitrogen ratios on biofloc production and biochemical composition and subsequent effects on the growth, physiological status and disease resistance of African catfish (*Clarias gariepinus*) cultured in glycerol-based biofloc systems. *Aquaculture* 483, 120–130. 10.1016/j.aquaculture.2017.10.016

[B11] Deng J. Zhang X. Lin B. Mi H. Zhang L. (2023). Excessive dietary soluble arabinoxylan impairs the intestinal physical and immunological barriers via activating MAPK/NF-κB signaling pathway in rainbow trout (*Oncorhynchus mykiss*). *Fish Shellfish Immunol.* 141:109041. 10.1016/j.fsi.2023.109041 37657558

[B12] Deng J. Zhang X. Sun Y. Mi H. Zhang L. (2021). Effects of different types of non-starch polysaccharides on growth, digestive enzyme activity, intestinal barrier function and antioxidant activity of rainbow trout (*Oncorhynchus mykiss*). *Aquacult. Rep.* 21:100864. 10.1016/j.aqrep.2021.100864

[B13] Detman A. Laubitz D. Chojnacka A. Kiela P. R. Salamon A. Barberán A. et al. (2021). Dynamics of dark fermentation microbial communities in the light of lactate and butyrate production. *Microbiome* 9:158. 10.1186/s40168-021-01105-x 34261525 PMC8281708

[B14] Douglas G. M. Maffei V. J. Zaneveld J. Yurgel S. N. Brown J. R. Taylor C. M. et al. (2020). PICRUSt2: An improved and extensible approach for metagenome inference. *Nat. Biotechnol.* 38, 685–688. 10.1038/s41587-020-0548-6 32483366 PMC7365738

[B15] Enjalbert F. Combes S. Zened A. Meynadier A. (2017). Rumen microbiota and dietary fat: A mutual shaping. *J. Appl. Microbiol.* 123, 782–797. 10.1111/jam.13501 28557277

[B16] FAO (2024). *The State of World Fisheries and Aquaculture 2024: Blue Transformation in Action.* Italy: Rome.

[B17] Fierer N. Bradford M. A. Jackson R. B. (2007). Toward an ecological classification of soil bacteria. *Ecology* 88, 1354–1364. 10.1890/05-1839 17601128

[B18] Fuirst M. Ward C. S. Schwaner C. Diana Z. Schultz T. F. Rittschof D. (2021). Compositional and functional microbiome variation between tubes of an intertidal polychaete and surrounding marine sediment. *Front. Mar. Sci.* 8:656506. 10.3389/fmars.2021.656506

[B19] Ge J. Bai N. Yu Y. Wang H. Qi Z. Zhang Z. et al. (2026). Effect of dietary protein/carbohydrate ratio on growth, digestion, and intestinal development in *Apostichopus japonicus*: Underlying molecular mechanisms. *Aquac. Rep.* 46:103338. 10.1016/j.aqrep.2025.103338

[B20] Gratz S. Hazim S. Richardson A. Scobbie L. Johnstone A. Fyfe C. et al. (2019). Dietary carbohydrate rather than protein intake drives colonic microbial fermentation during weight loss. *Eur. J. Nutr.* 58, 1147–1158. 10.1007/s00394-018-1629-x 29464347 PMC6499751

[B21] Guo H. Huang L. Hu S. Chen C. Huang X. Liu W. et al. (2020). Effects of carbon/nitrogen ratio on growth, intestinal microbiota and metabolome of shrimp (*Litopenaeus vannamei*). *Front. Microbiol.* 11:652. 10.3389/fmicb.2020.00652 32351483 PMC7176362

[B22] Guo M. Peng R. Jin K. Zhang X. Mo H. Li X. et al. (2024). Effects of Aeromonas infection on the immune system, physical barriers and microflora structure in the intestine of juvenile grass carp (*Ctenopharyngodon idella*). *Fish Shellfish Immunol.* 153:109790. 10.1016/j.fsi.2024.109790 39059563

[B23] Han Z. Ge L. Wen S. Sun J. (2023). Dysfunction of the intestinal physical barrier in the intestinal inflammation of tongue sole, *Cynoglossus semilaevis*, induced by *Shewanella algae* infection. *Fish Shellfish Immunol.* 139:108900. 10.1016/j.fsi.2023.108900 37315911

[B24] Hashish E. Merwad A. Elgaml S. Amer A. Kamal H. Elsadek A. et al. (2018). *Mycobacterium marinum infection in fish and man: Epidemiology, pathophysiology and management; a review*. *Vet. Q.* 38, 35–46. 10.1080/01652176.2018.1447171 29493404 PMC6831007

[B25] Huang B. Zhang S. Dong X. Chi S. Yang Q. Liu H. et al. (2022). Effects of fishmeal replacement by black soldier fly on growth performance, digestive enzyme activity, intestine morphology, intestinal flora and immune response of pearl gentian grouper (*Epinephelus fuscoguttatus* ♀ × *Epinephelus lanceolatus* ♂). *Fish Shellfish Immunol.* 120, 497–506. 10.1016/j.fsi.2021.12.027 34942373

[B26] Huang B. Zhang S. Dong X. Chi S. Yang Q. Liu H. et al. (2025). Dietary sodium humate improves intestinal mucosal immune and biological barriers of genetically improved farmed tilapia (*Oreochromis niloticus*). *Aquacult. Nutr.* 2025:3398904. 10.1155/anu/3398904 41112499 PMC12530943

[B27] Huang X. Aalto S. L. Dalsgaard J. Pedersen P. (2024). Can dietary C:N ratio influence water quality and microbiology in recirculating aquaculture systems? *Aquacult. Int.* 32, 7789–7805. 10.1007/s10499-024-01541-0

[B28] Huang X. Zhong L. Kang Q. Liu S. Feng Y. Geng Y. et al. (2021). A high starch diet alters the composition of the intestinal microbiota of largemouth bass *Micropterus salmoides*, which may be associated with the development of enteritis. *Front. Microbiol.* 12:696588. 10.3389/fmicb.2021.696588 34305862 PMC8297414

[B29] Huyben D. Roehe B. K. Bekaert M. Ruyter B. Glencross B. (2020). Dietary lipid: Protein ratio and n-3 long-chain polyunsaturated fatty acids alters the gut microbiome of Atlantic salmon under hypoxic and normoxic conditions. *Front. Microbiol.* 11:589898. 10.3389/fmicb.2020.589898 33424792 PMC7785582

[B30] Ingerslev H. C. Lunder T. Nielsen M. E. (2010). Inflammatory and regenerative responses in salmonids following mechanical tissue damage and natural infection. *Fish Shellfish Immunol.* 29, 440–450. 10.1016/j.fsi.2010.05.002 20472069

[B31] Kafilzadeh F. Dehdari F. (2015). Amylase activity of aquatic actinomycetes isolated from the sediments of mangrove forests in south of Iran. *Egypt. J. Aquat. Res.* 41, 197–201. 10.1016/j.ejar.2015.04.003

[B32] Larsen A. M. Mohammed H. H. Arias C. R. (2014). Characterization of the gut microbiota of three commercially valuable warmwater fish species. *J. Appl. Microbiol.* 116, 1396–1404. 10.1111/jam.12475 24529218

[B33] Ley R. E. Hamady M. Lozupone C. Turnbaugh P. J. Ramey R. R. Bircher J. S. et al. (2008). Evolution of mammals and their gut microbes. *Science* 320, 1647–1651. 10.1126/science.1155725 18497261 PMC2649005

[B34] Li M. Liang H. Yang H. Ding Q. Xia R. Chen J. et al. (2024). Deciphering the gut microbiome of grass carp through multi-omics approach. *Microbiome* 12:2. 10.1186/s40168-023-01715-7 38167330 PMC10763231

[B35] Li X. Yu Y. Feng W. Yan Q. Gong Y. (2012). Host species as a strong determinant of the intestinal microbiota of fish larvae. *J. Microbiol.* 50, 29–37. 10.1007/s12275-012-1340-1 22367934

[B36] Limborg M. T. Alberdi A. Kodama M. Roggenbuck M. Kristiansen K. Gilbert M. T. P. (2018). Applied hologenomics: Feasibility and potential in aquaculture. *Trends Biotechnol.* 36, 252–264. 10.1016/j.tibtech.2017.12.006 29395346

[B37] Liu W. Li E. Xu C. Chen L. Wang X. (2025). Nutritional strategies for Nile tilapia: Protein and carbohydrate balances in saline-alkaline aquaculture. *J. Anim. Sci. Biotechnol.* 16:86. 10.1186/s40104-025-01215-8 40524257 PMC12172231

[B38] Liu X. Xi Q. Yang L. Li H. Jiang Q. Shu G. et al. (2011). The effect of dietary *Panax ginseng* polysaccharide extract on the immune responses in white shrimp, *Litopenaeus vannamei*. *Fish Shellfish Immunol.* 30, 495–500. 10.1016/j.fsi.2010.11.018 21129487

[B39] Livak K. J. Schmittgen T. D. (2001). Analysis of relative gene expression data using real-time quantitative PCR and the 2^–ΔΔCT^ method. *Methods* 25, 402–408. 10.1006/meth.2001.1262 11846609

[B40] Lou M. Jing C. Liu X. Feng Y. Li X. (2025). Intestinal inflammation, dysfunction of intestinal digestion, and disorder in the intestinal microbiota and their metabolites caused by oral microcystis exposure in common carp (*Cyprinus carpio*). *Biology* 15:38. 10.3390/biology15010038 41514879 PMC12785017

[B41] Louca S. Parfrey L. W. Doebeli M. (2016). Decoupling function and taxonomy in the global ocean microbiome. *Science* 353, 1272–1277. 10.1126/science.aaf4507 27634532

[B42] Louis P. Flint H. J. (2017). Formation of propionate and butyrate by the human colonic microbiota. *Environ. Microbiol.* 19, 29–41. 10.1111/1462-2920.13589 27928878

[B43] Luan Y. Li M. Zhou W. Yao Y. Yang Y. Zhang Z. et al. (2023). The fish microbiota: Research progress and potential applications. *Engineering* 29, 137–146. 10.1016/j.eng.2022.12.011

[B44] Martin M. (2011). Cutadapt removes adapter sequences from high-throughput sequencing reads. *EMBnet J.* 17, 10–12. 10.14806/ej.17.1.200

[B45] Miao L. Hu F. C. Qiao F. Du Z. Y. Zhang M. L. (2020). Sodium acetate alleviated high-carbohydrate induced intestinal inflammation by suppressing MAPK and NF-κB signaling pathways in Nile tilapia (*Oreochromis niloticus*). *Fish Shellfish Immunol.* 98, 758–765. 10.1016/j.fsi.2019.11.024 31730927

[B46] Mouginot C. Kawamura R. Matulich K. L. Berlemont R. Allison S. D. Amend A. S. et al. (2014). Elemental stoichiometry of Fungi and Bacteria strains from grassland leaf litter. *Soil Biol. Biochem.* 76, 278–285. 10.1016/j.soilbio.2014.05.011

[B47] Nyholm L. Odriozola I. Bideguren G. M. Aizpurua O. Alberdi A. (2022). Gut microbiota differences between paired intestinal wall and digesta samples in three small species of fish. *PeerJ* 10:e12992. 10.7717/peerj.12992 35223211 PMC8877339

[B48] Osburn E. D. Weissman J. Strickland M. S. Bahram M. Stone B. W. McBride S. G. (2025). Relative abundances of bacterial phyla are strong indicators of community-scale microbial growth rates in soil. *Environ. Microbiome* 20:131. 10.1186/s40793-025-00790-7 41088467 PMC12522710

[B49] Ou W. Yu G. Zhang Y. Mai K. (2021). Recent progress in the understanding of the gut microbiota of marine fishes. *Mar. Life Sci. Technol.* 3, 434–448. 10.1007/s42995-021-00094-y 37073265 PMC10077274

[B50] Özel O. T. Çakmak E. Coşkun İ Çankırılıgil E. C. (2018). Evaluation of growth performance and intestine villi morphology of black sea trout (*Salmo labrax* Pallas, 1814) fed with different protein levels containing diets. *Ege J. Fish. Aquat. Sci.* 35, 125–130. 10.12714/egejfas.2018.35.2.04

[B51] Qi B. Zhang K. Qin S. Lyu D. He J. (2022). Glucose addition promotes C fixation and bacteria diversity in C-poor soils, improves root morphology, and enhances key N metabolism in apple roots. *PLoS One* 17:e0262691. 10.1371/journal.pone.0262691 35045112 PMC8773054

[B52] Qi X. Zhang Y. Zhang Y. Luo F. Song K. Wang G. et al. (2023). Vitamin B(12) produced by *Cetobacterium* somerae improves host resistance against pathogen infection through strengthening the interactions within gut microbiota. *Microbiome* 11:135. 10.1186/s40168-023-01574-2 37322528 PMC10268390

[B53] Reda R. M. Maricchiolo G. Quero G. M. Basili M. Aarestrup F. M. Pansera L. et al. (2022). Rice protein concentrate as a fish meal substitute in *Oreochromis niloticus*: Effects on immune response, intestinal cytokines, Aeromonas veronii resistance, and gut microbiota composition. *Fish Shellfish Immunol.* 126, 237–250. 10.1016/j.fsi.2022.05.048 35654384

[B54] Reese A. T. Pereira F. C. Schintlmeister A. Berry D. Wagner M. Hale L. P. et al. (2018). Microbial nitrogen limitation in the mammalian large intestine. *Nat. Microbiol.* 3, 1441–1450. 10.1038/s41564-018-0267-7 30374168 PMC6264799

[B55] Ruiz A. Gisbert E. Andree K. B. (2024). Impact of the diet in the gut microbiota after an inter-species microbial transplantation in fish. *Sci. Rep.* 14:4007. 10.1038/s41598-024-54519-6 38369563 PMC10874947

[B56] Sakai M. Hikima J.-I. Kono T. (2021). Fish cytokines: Current research and applications. *Fish. Sci.* 87, 1–9. 10.1007/s12562-020-01476-4

[B57] Shi F. Zou D. Zhang L. Guo N. Yu J. Degen A. A. et al. (2025). Increased urea nitrogen salvaging by a remodeled gut microbiota helps nonhibernating pikas maintain protein homeostasis during winter. *PLoS Biol.* 23:e3003436. 10.1371/journal.pbio.3003436 41100443 PMC12530534

[B58] Shin Y. Han S. Kwon J. Ju S. Choi T. G. Kang I. et al. (2023). Roles of short-chain fatty acids in inflammatory bowel disease. *Nutrients* 15:4466. 10.3390/nu15204466 37892541 PMC10609902

[B59] Sinsabaugh R. L. Manzoni S. Moorhead D. L. Richter A. (2013). Carbon use efficiency of microbial communities: Stoichiometry, methodology and modelling. *Ecol. Lett.* 16, 930–939. 10.1111/ele.12113 23627730

[B60] Soh M. Tay Y. C. Lee C. S. Low A. Orban L. Jaafar Z. et al. (2024). The intestinal digesta microbiota of tropical marine fish is largely uncultured and distinct from surrounding water microbiota. *NPJ Biofilms Microbiomes* 10:11. 10.1038/s41522-024-00484-x 38374184 PMC10876542

[B61] Su X. Ji D. Yao J. Zou Y. Yan M. (2022). Comparative analysis of intestinal characteristics of largemouth bass (*Micropterus salmoides*) and intestinal flora with different growth rates. *Fishes* 7:65. 10.3390/fishes7020065

[B62] Talwar C. Nagar S. Lal R. Negi R. K. (2018). Fish gut microbiome: Current approaches and future perspectives. *Indian J. Microbiol.* 58, 397–414. 10.1007/s12088-018-0760-y 30262950 PMC6141390

[B63] Villasante A. Ramírez C. Catalán N. Opazo R. Dantagnan P. Romero J. (2019). Effect of dietary carbohydrate-to-protein ratio on gut microbiota in Atlantic salmon (*Salmo salar*). *Animals* 9:89. 10.3390/ani9030089 30862122 PMC6466077

[B64] Villasante A. Ramírez C. Rodríguez H. Dantagnan P. Hernández A. Figueroa E. et al. (2022). Dietary carbohydrate-to-protein ratio influences growth performance, hepatic health and dynamic of gut microbiota in atlantic salmon (*Salmo salar*). *Anim. Nutr.* 10, 261–279. 10.1016/j.aninu.2022.04.003 35785253 PMC9234083

[B65] Wang A. Zhang Z. Ding Q. Yang Y. Bindelle J. Ran C. et al. (2021). Intestinal *Cetobacterium* and acetate modify glucose homeostasis via parasympathetic activation in zebrafish. *Gut Microbes* 13, 1–15. 10.1080/19490976.2021.1900996 33840371 PMC8043178

[B66] Wang G. Yu E. Xie J. Yu D. Li Z. Luo W. et al. (2015). Effect of C/N ratio on water quality in zero-water exchange tanks and the biofloc supplementation in feed on the growth performance of crucian carp, *Carassius auratus*. *Aquaculture* 443, 98–104. 10.1016/j.aquaculture.2015.03.015

[B67] Wang J. Lan K. Wu G. Wang Y. Zhou C. Lin H. et al. (2022). Effect of dietary carbohydrate level on growth, feed utilization, energy retention, body composition, and digestive and metabolic enzyme activities of juvenile cobia, *Rachycentron canadum*. *Aquacult. Rep.* 25:101211. 10.1016/j.aqrep.2022.101211

[B68] Wang Y. Lu Y. Zhang Y. Ning Z. Li Y. Zhao Q. et al. (2015). The draft genome of the grass carp (*Ctenopharyngodon idellus*) provides insights into its evolution and vegetarian adaptation. *Nat. Genet.* 47, 625–631. 10.1038/ng.3280 25938946

[B69] Wilson R. P. (1994). Utilization of dietary carbohydrate by fish. *Aquaculture* 124, 67–80. 10.1016/0044-8486(94)90363-8

[B70] Xue H. Guo J. You Y. Yin M. Ren C. Zhan J. et al. (2018). A fast and accurate way to determine short chain fatty acids in mouse feces based on GC–MS. *J. Chromatogr. B* 1099, 73–82. 10.1016/j.jchromb.2018.09.013 30243116

[B71] Zaki F. M. Said M. M. Amer M. Khalil R. H. Dighiesh H. S. (2025). Evaluation of biofloc system effects on water quality, growth, innate immunity, physiological status, and immune-and growth-related gene expressions in early growth stages of thin-lipped mullet (*Liza ramada*). *Aquac. Int.* 33:71. 10.1007/s10499-024-01696-w

[B72] Zhang X. Zhang J. Wang H. Lin B. Chen L. Li G. et al. (2019). Evaluation of soybean meal as alternative to fish meal in diet for juvenile Asian red-tailed catfish (*Hemibagrus wyckioides*). *Aquacult. Nutr.* 25, 1036–1049. 10.1111/anu.12921 PMC9973221 36860970

[B73] Zhao Z. Xu Q. Luo L. Wang C. A. Li J. Wang L. (2014). Effect of feed C/N ratio promoted bioflocs on water quality and production performance of bottom and filter feeder carp in minimum-water exchanged pond polyculture system. *Aquaculture* 434, 442–448. 10.1016/j.aquaculture.2014.09.006

[B74] Zimmerman A. E. Allison S. D. Martiny A. C. (2014). Phylogenetic constraints on elemental stoichiometry and resource allocation in heterotrophic marine bacteria. *Environ. Microbiol.* 16, 1398–1410. 10.1111/1462-2920.12329 24237481

