# Supplementary material for: Diet formulations with graded C:N ratios are associated with intestinal microbiota and health responses in GIFT tilapia (Oreochromis niloticus)
Source: Front Microbiol. 2026 Oct 8;17:1918261. doi: 10.3389/fmicb.2026.1918261 (PMC13686886; doi:10.3389/fmicb.2026.1918261)
Supplement: Supplementary file 1 [file Table_1.docx]

Supplementary Table S1. Effective library sizes and library-size sensitivity analyses for Bray-Curtis/PERMANOVA: per-sample effective library sizes.

| Sample | Effective library size |
| --- | --- |
| CN7.3_2 | 67,180 |
| CN7.3_3 | 66,511 |
| CN7.3_1 | 64,387 |
| CN8.1_2 | 60,091 |
| CN8.1_3 | 62,457 |
| CN8.1_1 | 59,188 |
| CN9.2_2 | 50,983 |
| CN9.2_1 | 61,505 |
| CN9.2_3 | 60,765 |
| CN10.7_2 | 58,700 |
| CN10.7_3 | 51,947 |
| CN10.7_1 | 60,178 |
| CN12.2_1 | 61,810 |
| CN12.2_2 | 60,769 |
| CN12.2_3 | 64,305 |
| CN14.5_2 | 62,882 |
| CN14.5_3 | 63,024 |
| CN14.5_1 | 63,019 |

Supplementary Table S2. Effective library sizes and library-size sensitivity analyses for Bray-Curtis/PERMANOVA: PERMANOVA sensitivity to library-size handling

| Analysis | Library-size handling | R² | Pseudo-F | P |
| --- | --- | --- | --- | --- |
| Primary | None; raw filtered ASV counts | 0.5735 | 3.227 | 0.0007 |
| TSS sensitivity | Total-sum scaling to relative abundance | 0.5713 | 3.198 | 0.0006 |
| Rarefaction sensitivity | Rarefied without replacement to 50,983 counts/sample | 0.5717 | 3.203 | 0.0006 |

Note: ASV, amplicon sequence variant; TSS, total-sum scaling. All PERMANOVA tests were based on Bray-Curtis dissimilarities and 9,999 unrestricted permutations. The rarefaction depth was the minimum effective library size observed in the final filtered ASV count matrix.

Supplementary Table S3. ANCOM-BC2 compositional sensitivity analysis of all taxa shown in Table 9.

|  |  | | ANCOM-BC2 | | | | | |
| --- | --- | --- | --- | --- | --- | --- | --- | --- |
| Items | | Primary signal in Table 9 | Global q | Linear LFC | Linear q | Quadratic LFC | Quadratic q |  |
| **Phylum-level** | |  |  |  |  |  |  |  |
| Fusobacteriota (%) | | Global; Linear; Quadratic | < 0.001 | -0.296 | 0.405 | 0.684 | 0.075 |  |
| Firmicutes (%) | | None | 0.003 | 0.310 | 0.304 | -0.183 | 0.408 |  |
| Proteobacteria (%) | | Global; Quadratic | 0.004 | -0.643 | 0.194 | -0.288 | 0.358 |  |
| Actinobacteriota (%) | | Global; Linear | < 0.001 | 1.573 | < 0.001 | -0.305 | 0.279 |  |
| Bacteroidota (%) | | None | < 0.001 | -1.097 | 0.215 | -1.272 | 0.231 |  |
| Chloroflexi (%) | | None | 0.032 | 0.100 | 0.745 | -0.386 | 0.279 |  |
| Cyanobacteria (%) | | None | 0.002 | 0.562 | 0.194 | -0.149 | 0.558 |  |
| Desulfobacterota (%) | | None | 0.008 | -0.972 | 0.215 | -0.630 | 0.358 |  |
| Verrucomicrobiota (%) | | None | < 0.001 | -0.227 | 0.633 | -1.046 | 0.075 |  |
| **Genus-level** | |  |  |  |  |  |  |  |
| *Cetobacterium* (%) | | Global; Linear; Quadratic | < 0.001 | -0.296 | 0.520 | 0.684 | 0.142 |  |
| *Mycobacterium* (%) | | Global; Linear | < 0.001 | 1.249 | 0.007 | -0.397 | 0.342 |  |
| *Dietzia* (%) | | Global; Linear | < 0.001 | 4.460 | < 0.001 | 1.029 | 0.318 |  |
| *Clostridium* (s.s 1) (%) | | None | 0.243 | 0.095 | 0.832 | 0.480 | 0.342 |  |
| Caldilineaceae (uncl.) (%) | | None | 0.124 | -0.164 | 0.774 | -0.400 | 0.342 |  |
| *Clostridium* (s.s 12) (%) | | Linear | < 0.001 | 1.072 | 0.007 | -0.183 | 0.642 |  |
| *Nordella* (%) | | None | 0.217 | 0.077 | 0.859 | -0.201 | 0.608 |  |
| Cyanobacteriales (uncl.) (%) | | None | 0.009 | 0.526 | 0.240 | -0.052 | 0.915 |  |
| Cyanobacteriia (uncl.) (%) | | None | 0.038 | 0.249 | 0.582 | -0.234 | 0.608 |  |
| *Methylocystis* (%) | | None | 0.022 | 0.554 | 0.234 | -0.286 | 0.543 |  |

ANCOM-BC2 used the corrected final ASV count matrix for bias estimation and phylum- and genus-level count matrices for inference. Taxa with mean relative abundance >0.1% were retained (9 phyla and 34 genera), and BH-FDR correction was applied separately to global, linear, and quadratic tests within each rank using all retained taxa; only taxa shown in Table 9 are reported here. Linear and quadratic LFCs represent first- and second-degree orthogonal polynomial contrasts across CN7.3–CN14.5. Pseudo-count sensitivity analysis was enabled.
