# Supplementary material for: Diet formulations with graded C:N ratios are associated with intestinal microbiota and health responses in GIFT tilapia (Oreochromis niloticus)
Source: Front Microbiol. 2026 Oct 8;17:1918261. doi: 10.3389/fmicb.2026.1918261 (PMC13686886; doi:10.3389/fmicb.2026.1918261)
Supplement: Supplementary file 2 [file Data_Sheet_3.zip › workflow.docx]

# 16s rRNA Study-specific workflow

## textual flow description

→ Intestinal contents
→ genomic DNA extraction and quantification
→ V3–V4 amplification with two-round PCR
→ library preparation and Illumina NovaSeq 6000 sequencing
→ quality filtering with Trimmomatic
→ primer removal with Cutadapt
→ paired-end read merging with USEARCH
→ chimera removal with UCHIME
→ ASV inference with DADA2 in QIIME 2
→ low-abundance ASV filtering
→ taxonomic assignment against SILVA
→ alpha- and beta-diversity analyses
→ PCoA and PERMANOVA
→ Bray–Curtis/PERMANOVA library-size sensitivity analysis
→ PERMDISP using betadisper and permutest
→ PICRUSt2 and FAPROTAX functional prediction
→ primary relative-abundance analysis using one-way ANOVA and orthogonal polynomial contrasts
→ ANCOM-BC2 compositional sensitivity analysis

## 1. DNA extraction, PCR amplification, and sequencing

Genomic DNA was extracted from intestinal-content samples using a VAMNE Stool/Soil DNA Extraction Kit (Vazyme, China). DNA concentrations were quantified using 1× dsDNA HS Working Solution (Yeasen, Shanghai, China) with a Synergy HTX multi-mode reader (BioTek, Santa Clara, CA, USA).

The V3–V4 region of the bacterial 16S rRNA gene was amplified using the forward primer ACTCCTACGGGAGGCAGCA and the reverse primer GGACTACHVGGGTWTCTAAT. PCR amplification was performed in two rounds. The first-round PCR was conducted using KOD FX Neo polymerase (No. KFX-201S; Toyobo, Osaka, Japan), and the resulting amplicons were purified using VAHTS DNA Clean Beads (Vazyme, Nanjing, China). No-template negative controls were included during amplification. No amplicon products were detected in the negative controls; therefore, these controls were not subjected to library construction or sequencing. The second-round PCR was conducted using Q5 High-Fidelity PCR Mix (New England Biolabs, Ipswich, MA, USA).

Following library quantification, paired-end sequencing was performed on an Illumina NovaSeq 6000 platform.

## 2. Raw-read quality filtering

Raw paired-end reads were initially quality-filtered using Trimmomatic v0.33. A sliding window of 50 bp was applied, and reads were trimmed when the average Phred quality score within the window decreased below 20. Other Trimmomatic filtering options were not retained in the archived project documentation.

## 3. Primer identification and removal

Forward and reverse primer sequences were identified and removed using Cutadapt v1.9.1. Primer matching allowed a maximum mismatch rate of 20% and required at least 80% coverage of the corresponding primer sequence.

The minimum-overlap value of 16 nt is the command-level implementation of the reported 80% primer-coverage criterion.

## 4. Paired-end read assembly

Primer-trimmed paired-end reads were assembled using USEARCH v10.0. A minimum overlap of 10 bp, a minimum similarity of 90% within the overlapping region, and a maximum of five mismatches were required.

## 5. Chimera removal

Chimeric sequences were identified and removed using UCHIME v8.1. Only non-chimeric merged reads were retained for subsequent ASV inference.

## 6. ASV inference

Non-chimeric merged reads were processed using the DADA2 method in QIIME 2 v2020.06 to denoise and dereplicate the sequences and generate amplicon sequence variants. DADA2 v1.18 was reported for this analysis.

## 7. Low-abundance ASV filtration

ASVs with a total abundance of fewer than two reads were removed before downstream analyses.

## 8. ASV identifier synchronization

The remaining ASVs were consecutively renumbered as ASV1, ASV2, ASV3, and so forth. The same identifier mapping was applied to the abundance table, representative-sequence FASTA file, taxonomy table, and all downstream files. A separate old-to-new ASV identifier mapping table was retained.

## 9. Taxonomic classification

Taxonomic classification was performed in QIIME 2 using the representative ASV sequences and the SILVA reference database, Release 138. A sequential classification procedure combining BLAST-based and Naive Bayes classifier-based methods was used.

**BLAST classification**

Representative ASV sequences were first classified using classify-consensus-blast. The minimum sequence identity was 90%, minimum query coverage was 90%, minimum consensus was 51%, and three accepted BLAST hits were considered for consensus assignment.

**Naive Bayes classification**

ASVs that did not receive a reference match from the BLAST-based procedure were subsequently classified using classify-sklearn, with a classifier-confidence threshold of 0.7.

**Profile summarization**

ASV abundances were aggregated according to their taxonomic assignments to obtain phylum- and genus-level taxonomic profiles, which were expressed as relative abundances. These relative-abundance data were used directly for subsequent statistical analyses without log-ratio or other compositional transformation.

## 10. Phylogenetic tree

Weighted and unweighted UniFrac distances were calculated using the rooted phylogenetic tree supplied with the archived study outputs (tree.nwk).

## 11. Alpha-diversity analysis

ACE, Shannon, and Simpson indices were calculated from the filtered ASV table using QIIME 2. No rarefaction or other library-size normalization was applied to the filtered ASV count table before alpha-diversity analysis. Among-treatment differences were evaluated using one-way ANOVA, and linear and quadratic trends across the six equally spaced ordered treatment levels were evaluated using first- and second-degree orthogonal polynomial contrasts, respectively.

## 12. Beta-diversity and PCoA

Beta diversity was assessed using Bray–Curtis, binary Jaccard, weighted UniFrac, and unweighted UniFrac distance matrices. Principal coordinates analysis was used to visualize the corresponding multivariate distance relationships.

Bray–Curtis and binary Jaccard distances were calculated directly from the same filtered ASV count table without rarefaction or other library-size normalization. Weighted and unweighted UniFrac distances additionally incorporated the rooted phylogenetic tree.

## 13. PERMANOVA

Differences in beta diversity among treatment groups were evaluated using PERMANOVA implemented with the Adonis function in the R package vegan. The Bray–Curtis PERMANOVA used the Bray–Curtis dissimilarity matrix described above, with treatment group as the explanatory variable. The final Bray–Curtis PERMANOVA was performed using 9,999 unrestricted permutations.

## 14. Library-size sensitivity analysis for Bray–Curtis/PERMANOVA

Effective library size was defined as the sum of ASV counts in the final filtered ASV count matrix. Two sensitivity analyses were performed using the same ASV matrix: Bray–Curtis/PERMANOVA after total-sum scaling to relative abundance and Bray–Curtis/PERMANOVA after rarefying all samples without replacement to the minimum effective library size.

## 15. PERMDISP / betadisper

Homogeneity of multivariate dispersion among treatment groups was assessed using PERMDISP, implemented with the betadisper function in the R package vegan. The analysis was based on the same Bray–Curtis dissimilarity matrix used for PERMANOVA. Distances from individual samples to the centroid of their respective treatment groups were calculated using type = "centroid". Differences in dispersion were evaluated using permutest with 9,999 unrestricted permutations.

## 16. PICRUSt2 functional prediction

Microbial gene-family and pathway profiles were predicted using PICRUSt2 v2.4.2 from the filtered ASV abundance table and corresponding representative ASV sequences. The analysis comprised phylogenetic placement of ASVs, hidden-state prediction of marker-gene and gene-family copy numbers, prediction of metagenomic gene-family abundance, and pathway inference.

## 17. FAPROTAX functional annotation

Taxonomy-based ecological and metabolic functions were inferred using FAPROTAX by mapping the taxonomic assignments of individual ASVs to literature-supported prokaryotic functional categories.

## 18. Statistical analysis of taxonomic and predicted functional profiles

Phylum- and genus-level relative abundances and PICRUSt2- and FAPROTAX-predicted functional relative abundances were analyzed directly without transformation. Among-treatment differences were evaluated using one-way ANOVA. Linear and quadratic trends across CN7.3, CN8.1, CN9.2, CN10.7, CN12.2, and CN14.5, treated as six equally spaced ordered levels, were evaluated using first- and second-degree orthogonal polynomial contrasts within the one-way ANOVA framework, respectively.

For the primary phylum- and genus-level relative-abundance analysis, an executable R script is provided that reproduces taxonomic aggregation from the corrected ASV matrix, relative-abundance calculation, the >0.1% overall mean abundance filter, one-way ANOVA, orthogonal polynomial trend contrasts, and BH-FDR correction.

## 19. Multiple-testing correction for relative-abundance analyses

For the primary analyses of phylum- and genus-level relative abundances, taxa with an overall mean relative abundance >0.1% were included in multiple-testing correction. Overall mean relative abundance was calculated by first dividing each taxon count by the total count in the final filtered ASV matrix for the corresponding sample and then averaging the resulting relative abundances across all 18 samples. Benjamini–Hochberg false discovery rate (BH-FDR) correction was applied separately to one-way ANOVA, linear-trend, and quadratic-trend *P* values within each data domain (phylum, genus, PICRUSt2, and FAPROTAX).

## 20. ANCOM-BC2 compositional sensitivity analysis

To assess the robustness of the primary taxonomic relative-abundance findings to compositional effects, ANCOM-BC2 (ANCOMBC v2.14.0) was rerun from the corrected final 18-sample ASV count matrix. The complete final ASV count matrix was supplied as the lowest-level data input for estimation of sampling fractions and bias correction. ASV counts were independently aggregated to the phylum and genus levels. For abundance filtering, sample-specific relative abundance was calculated using the total library size of the corrected final ASV count matrix as the denominator, and the arithmetic mean across all 18 samples was then calculated for each taxon. Taxa with an overall mean relative abundance >0.1% were retained, yielding 9 phyla and 34 genera. Non-informative root-level labels (NA/blank, Unknown, Unassigned, Other, and unclassified_Bacteria) were excluded, whereas informative lower-level unclassified taxa were retained.

The filtered phylum- and genus-level count matrices were supplied as aggregate_data. The global treatment test was fitted using fix_formula = "Treatment", group = "Treatment", and global = TRUE, with treatment represented as a six-level categorical factor. Linear and quadratic effects were evaluated in a separate ANCOM-BC2 model using fix_formula = "TrendLinear + TrendQuadratic", group = NULL, and global = FALSE. The two trend variables were first- and second-degree orthogonal polynomial contrasts generated from equally spaced treatment-order codes 1–6 corresponding to CN7.3, CN8.1, CN9.2, CN10.7, CN12.2, and CN14.5, respectively. The ANCOM-BC2 built-in constrained trend test (trend = TRUE) was not used.

BH adjustment (p_adj_method = "BH") was performed separately for the phylum/global, phylum/linear, phylum/quadratic, genus/global, genus/linear, and genus/quadratic testing families. Other analysis settings were rand_formula = NULL, prv_cut = 0, lib_cut = 0, s0_perc = 0.05, struc_zero = FALSE, neg_lb = FALSE, alpha = 0.05, and pseudo_sens = TRUE. Taxa with BH-adjusted q < 0.05 were considered statistically significant. For conclusions described as robust to the ANCOM-BC2 pseudo-count sensitivity analysis, significance additionally required passed_ss = TRUE. The complete analysis specification, design matrices, FDR-family membership, raw and adjusted test results, session information, and executable R script are provided in Supplementary Data Sheet 2 and the accompanying analysis files.

## Study-specific outputs

Files included in the submitted supplementary package:

filtered ASV abundance table;

representative ASV sequences in FASTA format;

separate taxonomy table linked by ASV identifier;

SRA/sample metadata; and this study-specific workflow.

## Reproducibility Statement

The corrected final ASV abundance table, representative ASV sequences, taxonomy information, sample metadata, and the study-specific workflow are provided with the supplementary materials. For the revision-stage analyses, executable R scripts are additionally provided for the primary phylum- and genus-level relative-abundance analysis and the ANCOM-BC2 compositional sensitivity analysis. The primary relative-abundance script reproduces taxonomic aggregation, relative-abundance calculation, abundance filtering, one-way ANOVA, first- and second-degree orthogonal polynomial contrasts, and BH-FDR correction directly from the corrected final ASV count matrix. The ANCOM-BC2 script regenerates the phylum- and genus-level abundance-filtering families and the corresponding global, linear, and quadratic ANCOM-BC2 analyses from the same corrected count matrix.

Complete command-by-command scripts for some upstream sequencing-processing steps were not retained in the archived project documentation because portions of the original upstream pipeline were implemented by a collaborating commercial service provider. Accordingly, the workflow documents the upstream analytical stages, software versions, and parameters that could be verified from the archived records, whereas the revision-stage primary relative-abundance and ANCOM-BC2 analyses are provided as independently executable and reproducible analyses.
